# Outperformance in Acrylation: Supported D-Glucose-Based Ionic Liquid Phase on MWCNTs for Immobilized Lipase B from *Candida antarctica* as Catalytic System

**DOI:** 10.3390/ma14113090

**Published:** 2021-06-04

**Authors:** Anna Szelwicka, Karol Erfurt, Sebastian Jurczyk, Slawomir Boncel, Anna Chrobok

**Affiliations:** 1 Department of Chemical Organic Technology and Petrochemistry, Silesian University of Technology, Krzywoustego 4, 44-100 Gliwice, Poland; Anna.Szelwicka@polsl.pl (A.S.); karol.erfurt@polsl.pl (K.E.); 2 Institute for Engineering of Polymer Materials and Dyes, Lukasiewicz Research Network, Sklodowskiej-Curie 55, 87-100 Torun, Poland; sebastian.jurczyk@impib.lukasiewicz.gov.pl; 3 Department of Organic Chemistry, Bioorganic Chemistry and Biotechnology, Silesian University of Technology, Krzywoustego 4, 44-100 Gliwice, Poland

**Keywords:** alkyl acrylates, esterification, biocatalysis, supported ionic liquid phase, lipases

## Abstract

This study presents a highly efficient method of a synthesis of *n*-butyl acrylate via esterification of acrylic acid and *n*-butanol in the presence of supported ionic liquid phase (SILP) biocatalyst consisting of the lipase B from *Candida antarctica* (CALB) and multi-walled carbon nanotubes (MWCNTs) modified by D-glucose-based ionic liquids. Favorable reaction conditions (acrylic acid: *n*-butanol molar ratio 1:2, cyclohexane as a solvent, biocatalyst 0.150 g per 1 mmol of acrylic acid, temperature 25 °C) allowed the achievement of a 99% yield of *n*-butyl acrylate in 24 h. Screening of various ionic liquids showed that the most promising result was obtained if *N*-(6-deoxy-1-*O*-methoxy-α-D-glucopyranosyl)-*N*,*N*,*N*-trimethylammonium bis-(trifluoromethylsulfonyl)imide ([N(CH_3_)_3_GlcOCH_3_][N(Tf)_2_]) was selected in order to modify the outer surface of MWCNTs. The final SILP biocatalyst–CNTs-[N(CH_3_)_3_GlcOCH_3_][N(Tf)_2_]-CALB contained 1.8 wt.% of IL and 4.2 wt.% of CALB. Application of the SILP biocatalyst led to the enhanced activity of CALB in comparison with the biocatalyst prepared via physical adsorption of CALB onto MWCNTs (CNTs-CALB), as well as with commercially available Novozyme 435. Thus, the crucial role of IL in the stabilization of biocatalysts was clearly demonstrated. In addition, a significant stability of the developed biocatalytic system was confirmed (three runs with a yield of ester over 90%).

## 1. Introduction

Biomass conversion to value-added chemicals and fuels is nowadays one of the top challenges in green chemistry. The catalyst design is considered as the key approach to develop efficient, low-energy and environmentally friendly processes. On the other hand, biocatalysis is the preferable route for the synthesis of bio-based chemicals. Among them, bio-based acrylic acid (AA) and its esters (AEs) are interesting target molecules in the production of all-acrylic polymers, vinyl and styrene acrylic copolymers, adhesives, paints, coatings, ion-exchange resins, detergents, and flocculants [1,2,3].

Several routes for the AEs production are possible via bio-based and fossil sources [4]. Such sources include 3-hydroxypropionic acid, lactic acid, glycerol, or acetic acid. Nevertheless, industrial methods for the production of AEs are based on simple esterification of fossil-based AA, catalyzed by H_2_SO_4_ at 100 °C. This approach, however, suffers from the formation of by-products (AA dimers and trimers and alkyl alkoxy esters) [5]. The problems concerning the process equilibrium are also challenging. On the other hand, transesterification of short alkyl AEs with the longer chain alcohols could be convenient, since the strong acid can be replaced by inorganic complexes, e.g., LiCl/CaO [6]. However, a higher temperature (120 °C) still promotes polymerization of AEs.

Design and green synthesis of a superactive biocatalyst for the AA esterification—competitive to the industrial routes—is essential. Enzymes have already been used as biocatalysts for the synthesis of AEs, nevertheless mostly in transesterification. In fact, the interest in enzymatic transesterifications goes back to 1990 when transacrylation of esters in the presence of lipase from *Chromobacterium viscosum* was described [7]. The studies were continued using immobilized lipase B from *Candida antarctica* (CALB) (in its commercially available form—Novozyme 435 [8,9,10,11]) in those days of five times higher activity. Nonetheless, there are only two works describing direct esterification of AA in the presence of Novozyme 435: by *n*-octanol and methyl glucoside [12,13]. Therein, special attention was paid to AA concentration and the solvent choice as the key factors.

Most of the scientific data on the use of enzymes in the synthesis of AEs are enclosed in patents [14,15,16,17], which clearly confirms the industrial interest. Indeed, biocatalysis brings clear advantages over the chemical methods; hence, the former avoids acid/base catalysts and operates under mild conditions (30–60 °C) without the formation of by-products. To date, Novozyme 435 has showed the highest activity in the synthesis of AEs. However, for the industrial use, other important factors are rather fundamental. These are the thermal and chemical stability of the enzyme as well as its reuse in the consecutive cycles or adaptability with the flow system. It must be here emphasized that none of these characteristics have been so far presented for the enzymatic synthesis of AEs.

Several factors are known to stabilize enzymes [18,19]. One of the most important methods is their covalent and non-covalent (i.e., adsorption-based) immobilization [20,21] on solid supports of well-defined surface properties. Those processes might be beneficial in the light of enhanced activity, selectivity and/or specificity [22]. Here, multi-walled carbon nanotubes (MWCNTs) are excellent lipase supports due to the hydrophobicity of their outermost shells. Hence, a successful immobilization corresponds to the ‘interfacial activation’. Briefly, in the lipase in its open form, as the lid is mobile, the active centers become more available to the reactants. Under the favorable conditions, lipases can be immobilized on hydrophobic surfaces via the interfacial activation, fixing them in open conformations [23,24]. Nowadays, the progress achievable by applying hydrophobic MWCNTs for the immobilization of CALB is indisputable [20]. For instance, our previous studies have proved the high stability of MWCNTs-CALB hybrids (pristine and polytetrafluoroethylene-modified MWCNTs) with a consecutive high activity and stability in organic synthesis in both batch and flow systems [25,26,27,28,29].

Non-volatile ionic liquids (ILs) with tailor-made physicochemical properties are one of the most attractive modifiers of enzyme solid supports [30,31,32]. These fully ionic species, with a melting point below 100 °C, consist of bulky organic cations and organic or inorganic anions. [33,34]. The IL structure can be defined as the organized ‘nanostructure’, with hydrogen bonded polymeric supramolecules characterized by polar and non-polar regions [35,36]. The enzymes are known from their exceptional properties, inter alia, maintenance of the active conformations of the enzymes. For instance, CALB is compatible with hydrophobic ILs that result in the exposure of the catalytic triad of CALB to ILs and increase the overall stability and activity of the enzyme [37,38]. It was proven that the same IL can act otherwise in the two various reaction types, i.e., activating or deactivating [39].

Supported ionic liquid phase (SILP) materials for biocatalysis are based on the immobilization of the given, primary IL thin film on a solid support where the enzyme is later adsorbed. In fact, the final biocatalyst is solid, while the reaction can be carried out in the IL film. In designing SILP materials for the biocatalysis, the following IL properties are crucial: hydrophobicity, miscibility with solvents, polarity, nucleophilicity, hydrogen bonding ability, viscosity, enzyme dissolution, and the surfactant effect [30,40,41]. The other important factors characterizing SILP materials toward biocatalysis are IL loadings and the morphology of the support [30].

Only few works describe the use of MWCNTs for SILP biocatalysis [42,43]. ILs of various functional groups, such as alkyl, carboxyl, amino, or hydroxyl, were covalently tethered to the MWCNTs surface and further used for CALB adsorption. The catalytic performance SILP biocatalyst was demonstrated in the hydrolysis of triacetin. The activity and thermal stability of such SILP-enzyme hybrids were superior to conventional approaches and their reusability has been significantly improved. Nevertheless, leaching of the enzyme from the SILP carrier was the main factor responsible for the gradual decrease in the overall activity of the hybrids.

It is worth underlining that the use of a commercially available biocatalytic system, i.e., Novozyme 435, was proven to be poorly stable in the recycle tests due to thermal and mechanical deterioration, occasionally even after the first reaction [25,26,27,28,29]. Further, the main advantage of SILP materials is to minimize the amount of ILs compared to the catalysis in bulk. On the other hand, biodegradability and toxicity of ILs is often questionable. Herein, to tackle this problem, we propose the use of task-specific carbohydrate-based ILs, which were synthesized in our group while their applicability was already demonstrated *inter alia* as active organocatalysts, solvents, herbicides, N-doped materials, and conjugated polymers with the potential to be used as the neural interfaces and tissue scaffolds [44,45,46,47,48,49].

By employing the uniqueness of MWCNTs and IL-based systems in catalysis, we apply the proposed SILP material as the innovative solid support for CALB. In addition, biocatalysis perfectly fits to the idea of designing sustainable processes [50], which makes the proposed approach extremely attractive. Here, D-glucose-based ILs were used as the appealing and multi-functional MWCNTs modifiers. We demonstrate that morphological and physicochemical compatibilization of the enzyme with the bio-based SILP on MWCNTs brings superb cooperation toward the activity and stability of the biocatalytic systems. Hence, our idea fully conforms to sustainability in the chemical industry.

## 2. Materials and Methods

### 2.1. Materials

Acrylic acid, *n*-butanol, solvents, 1-butyl-3-methylimidazolium octylsulphate (purity 98%), 1-ethyl-3-methylimidazolium octylsulphate (purity 98%), 1-ethyl-3-methylimidazolium dioctylphosphate (purity 98%), 1-butyl-3-methylimidazolium dioctylphosphate (purity 98%), and *n*-decane were purchased from Chemat, Gdansk, Poland. Lipase B from *Candida antarctica* (activity 5000 U∙L∙kg^−1^), Novozyme 435 (biocatalyst consisting of lipase B from *Candida antarctica* immobilized on Lewatit VP OC 1600, activity 40,000 U∙L∙kg^−1^), methyl-α-D-glucopyranose (purity 98%), tetrabromomethane (purity 99%), triphenylphosphine (purity 99%), *N*,*N*,*N*-trimethylamine (purity 99.8%), *N*-butyl-*N*,*N*-dimethylamine (purity 99.8%), and lithium bis(trifluoromethylsulfonyl)imide (purity 99%) were purchased from Sigma-Aldrich (Merck Group, Warsaw, Poland). 1-Butyl-3-methylimidazolium tetrafluoroborate (purity 98%), 1-butyl-3-methylimidazolium bis(trifluoromethylsulfonyl)imide (purity 98%), 1-butyl-3-methylimidazolium dicyanamide (purity 98%), 1-ethyl-1-methylpyrrolidinium bis(trifluoromethylsulfonyl)imide (purity 99%), 1-ethyl-3-methylimidazolium ethylsulphate (purity 98%), 1-ethyl-3-methylimidazolium dimethylphosphate (purity 98%), and 1-butyl-3-ethylimidazolium chloride (purity 99%) were purchased from IoLiTech Ionic Liquids Technologies GmbH, Heilbronn, Germany. Industrial grade MWCNTs were purchased from CheapTubes Inc. (Grafton, VT, USA).

### 2.2. Methods

GC-FID analysis was performed using Shimadzu GC-2010 Plus equipped with a Zebron ZB5MSi column (30 m × 0.32 mm × 0.25 μm) (Phenomenex, Warsaw, Poland) (Supplementary Materials Section S1) and Agilent Technologies 7890C equipped with a mass spectrometer Agilent Technologies 5975C detector (Basel, Switzerland) with HP 5MS column (30 m × 0.25 mm × 0.25 μm) (Phenomenex, Warsaw, Poland) and electron impact (EI) ionization at 70 eV (Supplementary Materials Section S2).

High-resolution electrospray ionisation mass spectroscopy (Supplementary Materials MS) analysis was performed using a Waters Xevo G2 QTOF apparatus (Waters, Warsaw, Poland) (an injection system–cone voltage set at 50 V; source heat set up to 120 °C).

^1^H NMR (600 MHz) and ^13^C NMR (151 MHz) spectra were recorded on Varian system (Palo Alto, CA, USA) (Supplementary Materials Section S3).

Scanning electron microscopy (SEM) images were plotted utilizing a Phenom Pro desktop SEM instrument (Thermo Fischer Scientific, Warsaw, Poland) equipped with an EDS detector (15 kV) (Supplementary Materials Section S4).

TGA analysis was performed using Mettler Toledo TGA851e thermobalance; samples (10–15 mg) were heated from 25 to 800 °C and a temperature rate was set to 10 °C min^−1^; 70 μL Al_2_O_3_ crucibles were used as a standard, analyses were run under a dynamic nitrogen flow set at 60 mL/min (Supplementary Materials Section S5).

The presence of the lipase in the filtrate was checked using Lowry’s protein detection method according to the literature; 28 UV-VIS spectra were recorded using a Jasco V-650 spectrophotometer (Jasco, Cracow, Poland) (λ = 670 nm, 25 °C) (Supplementary Materials Section S6).

### 2.3. Synthetic Procedures

*Synthesis of methyl-6-bromo-6-deoxy-α-D-glucopyranose*: Into a three-necked round bottom flask, methyl-α-D-glucopyranose (104 mmol, 20.3 g) and 300 mL of pyridine were introduced. The mixture was cooled down to 0 °C in an ice bath. Next, CBr_4_ (150 mmol, 48.3 g) and PPh_3_ (200 mmol, 57.4 g) were added and stirred for 15 min. The reaction was carried out under a nitrogen atmosphere. Next, the mixture was heated up to 65 °C and stirred for 4 h (the mixture changed color from yellow to pale brown). Next, methanol (100 mL) was added in order to finish the reaction. Then, the reaction mixture was concentrated. Pyridine was evaporated after the addition of a few portions of toluene toward formation of the negative azeotrope. The product was purified using column chromatography (methanol: dichloromethane = 1:9 *v/v* and methanol: ethyl acetate = 1:9 *v*/*v*). Colorless solid was obtained in 72.9% yield.

*Synthesis of (a) N-(6-deoxy-1-O-methoxy-α-D-glucopyranosyl)-N,N,N-trimethylammonium bromide and (b) N-(6-deoxy-1-O-methoxy-α-D-glucopyranosyl)-N-butyl-N,N-dimethylammonium bromide*: To a closed steel reactor maintaining slight overpressure methyl-6-bromo-6-deoxy-α-D-glucopyranose (25 mmol, 6.37 g) and (a) 19.8 mL (223 mmol, 13.2 g) of N,N,N-trimethylamine in 40 mL of ethanol, or (b) 4.6 mL (33 mmol, 3.31 g) of N-butyl-N,N-dimethylamine in 30 mL of ethanol were introduced. The mixture was heated up to 70 °C and stirred for 68 h, maintaining slight overpressure. Then, the mixture was filtered off and a solid was washed with hexane (3 × 50 mL). Pale yellow solid products were obtained with (a) 90.7% yield and (b) 92.3% yield, respectively.

*Synthesis of (a) N-(6-deoxy-1-O-methoxy-α-D-glucopyranosyl)-N,N,N-trimethylammonium bis-(trifluoromethylsulfonyl)imide and (b) N-(6-deoxy-1-O-methoxy-α-D-glucopyranosyl)-N-butyl-N,N-dimethylammonium bis(trifluoromethylsulfonyl)imide*: Previously synthesised bromides (10 mmol) were dissolved in 10 mL of deionized water and, next, 10 mL of aqueous solution of lithium bis(trifluoromethylsulfonyl)imide (11 mmol, 3.16 g) (1.1 eq. in relation to the bromide) was added dropwise. The mixture was stirred at 25 °C for 1 h. After the reaction was finished, the post-reaction mixture was extracted with ethyl acetate (5 × 25 mL). The organic phase was concentrated under vacuum and washed with deionized water (2 × 20 mL), dried over anhydrous Na_2_SO_4,_ and evaporated under vacuum, obtaining yellow liquids with yields a) 65% and b) 63%, respectively.

*N-(6-deoxy-1-O-methoxy-α-D-glucopyranosyl)-N,N,N-trimethylammonium bis(trifluoromethylsulfonyl)imide:*

^1^H NMR (600 MHz, DMSO): δ/ppm = 5.45 (d, J = 6.0 Hz, 1H), 5.04 (d, J = 5.1 Hz, 1H), 4.93 (d, J = 6.4 Hz, 1H), 4.60 (d, J = 3.7 Hz, 1H), 3.57 (dd, J = 47.6, 10.9 Hz, 2H), 3.44–3.48 (m, 2H), 3.38 (s, 3H), 3.22–3.24 (m, 1H), 3.12 (s, 9H), 2.88–2.94 (m, 1H). ^13^C NMR (151 MHz, DMSO): δ/ppm = 121.16, 117.96, 100.82, 72.54, 71.74, 71.29, 67.15, 66.99, 56.56, 53.53. ESI-MS: [M^+^] calc.: 236.1498; found: 236.1500; [M^−^] calc.: 279.9173; found: 279.9176.

*N-(6-deoxy-1-O-methoxy-α-D-glucopyranosyl)-N-butyl-N,N-dimethylammonium bis(trifluoromethylsulfonyl)imide:*

^1^H NMR (600 MHz, DMSO): δ/ppm = 5.47 (d, J = 6.0 Hz, 1H), 5.05 (d, J = 5.1 Hz, 1H), 4.93 (d, J = 6.4 Hz, 1H), 4.59 (d, J = 3.7 Hz, 1H), 3.92–3.82 (m, 1H), 3.44–3.48 (m, 2H), 3.36 (s, 3H), 3.34–3.26 (m, 2H), 3.25–3.17 (m, 1H), 3.10–3.03 (m, 6H), 2.97–2.86 (m, 1H), 2.79–2.68 (m, 1H), 1.77–1.57 (m, 2H), 1.38–1.14 (m, 2H), 1.00–0.81 (m, 3H). ^13^C NMR (151 MHz, DMSO): δ/ppm = 121.10, 117.90, 100.83, 72.44, 71.69, 71.21, 66.73, 64.80, 64.01, 56.56, 51.35, 51.28, 23.71, 19.17, 13.46. ESI-MS: [M^+^] calc.: 278.1967; found: 278.1963; [M^−^] calc.: 279.9173; found: 279.9174.

*Synthesis of the CNTs-IL*: MWCNTs (1.00 g), acetonitrile (25 mL), and IL (0.5 g) were introduced into a 50-mL round-bottom flask. The suspension was stirred for 8 h at 25 °C using a magnetic stirrer (1000 rpm), the solid was filtered off under vacuum, washed with acetonitrile (5 × 20 mL), and dried on the Schlenk line (1 mbar, 25 °C) until the mass was constant.

*Synthesis of the CNTs-IL-CALB*: The CNTs-IL (1 g), deionized water (30 mL), and an aqueous-glycerine solution of CALB (7.5 g) were added into a 50 mL round-bottom flask. The flask contents were stirred in a thermostatic shaker at 25 °C for 3 h. Next, the CNTs-IL-CALB biocatalyst was filtered off under vacuum, washed with deionized water (2 × 30 mL), and dried in a desiccator over anhydrous P_2_O_5_ at 5 °C for 3 days.

*General procedure for the synthesis of the n-butyl acrylate*: CNTs-IL-CALB (0.150 g), cyclohexane (1 mL), acrylic acid (1 mmol, 0.072 g), *n*-decane (internal standard, 0.014 g), and *n*-butanol (0.149 g, 2 mmol) were introduced into a 10 mL round-bottom flask. The flask was sealed with a septum and mixed in a thermostatic shaker at 25 °C for 24 h. In order to determine the reaction progress, 20 μL samples were collected, diluted with 1.5 mL of acetonitrile and analyzed using a GC-FID apparatus. After accomplishing the reaction, the biocatalyst was filtered off. Next the distillation of filtrate gave *n*-butyl acrylate (10 mbar, 30 °C, yield 95%).

^1^H NMR (600 MHz, CDCl_3_) of *n*-butyl acrylate: δ/ppm = 6.51–6.22 (m, 1H), 6.06 (ddd, J = 17.3, 10.4, 1.0 Hz, 1H), 5.84–5.65 (m, 1H), 4.10 (td, J = 6.7, 0.7 Hz, 2H), 1.74–1.51 (m, 2H), 1.35 (dd, J = 15.1, 7.5 Hz, 2H), 0.89 (td, J = 7.4, 0.8 Hz, 3H). ^13^C NMR (151 MHz, CDCl_3_): δ/ppm = 166.54, 130.59, 128.93, 64.63, 30.94, 19.41, 13.94. GC-MS (EI) m/z (%): 128 (0) 113 (2), 99 (4), 85 (10), 73 (30), 55 (100), 41 (20), 27 (20), 15 (1).

*Recycle of the CNTs-IL-CALB biocatalyst:* CNTs-IL-CALB after the reaction was filtered off, washed with 30 mL of cyclohexane and dried under vacuum on a Schlenk line (24 h, <1 mbar, 25 °C), and used for another cycle of the reaction.

## 3. Results

### 3.1. Preliminary Studies

**Scheme 1:**
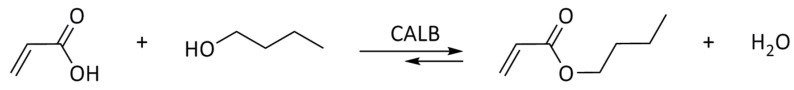
The model esterification reaction used in this study.

**Scheme 2:**
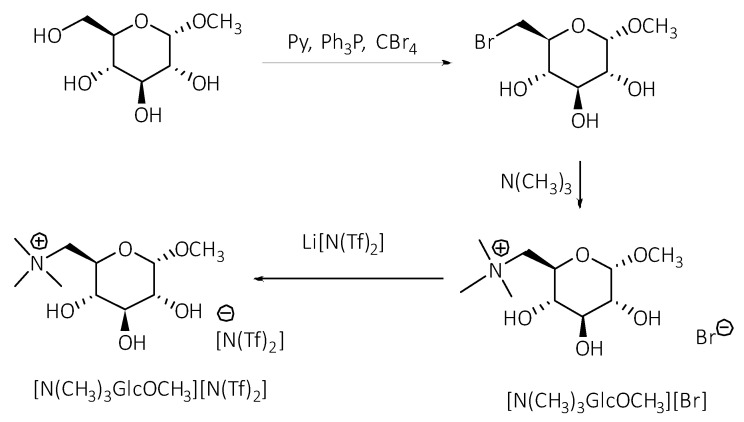
The synthesis of [N(CH_3_)_3_GlcOCH_3_][N(Tf)_2_].

In the preliminary studies, the comparative experiments using native CALB in the esterification of acrylic acid using various solvents were performed to show the activity of ILs in the model reaction. Synthesis of *n*-butyl acrylate (at 25 °C) was chosen as the model reaction (Scheme 1), as it is the largest-volume acrylate ester used in the production of all-acrylic, vinyl acrylic, and styrene acrylic co-polymers. The molar ratio of AA to *n*-BuOH was maintained at the level 1:2, while for each 1 mmol of AA, 0.100 g of CALB was used. The initial runs were performed in non-polar, aprotic classical solvent cyclohexane, which is favorable for CALB [25,26,27,28], and in ILs of various physicochemical properties: hydrophilic 1-butyl-3-methylimidazolium octylsulphate [bmim][OcSO_4_] and 1-butyl-3-methylimidazolium dioctylphosphate [bmim][Oc_2_PO_4_], as well as hydrophobic 1-butyl-3-methylimidazolium bis(trifluoromethylsulfonyl)imide [bmim][N(Tf)_2_]. Additionally, D-glucose-based IL, i.e., *N*-(6-deoxy-1-*O*-methoxy-α-D-glucopyranosyl)-*N*,*N*,*N*-trimethylammonium bis(trifluoromethylsulfonyl)imide [N(CH_3_)_3_GlcOCH_3_][N(Tf)_2_] (Scheme 2) was selected for the studies due to the presence of hydrogen bonding donor and acceptor centers. Carbohydrates are bio-based resources which can be applied in lieu of commonly used fossil-derived *fine chemicals*. Hence, this choice was motivated mostly by the environmental factors [51].

Bromide ILs of a quaternary ammonium core substituted with a D-glucose moiety at the anomeric carbon atom, and various length alkyl chains, were previously synthesized in our group. Such ILs showed no in vitro cytotoxicity against the promyelocytic leukemia rat cell line [44]. Here, we have pre-selected D-glucose-based ILs modified at the terminal carbon atom with bistriflimide counterion [N(Tf)_2_], which we used previously for the fabrication of nitrogen-doped carbons [49]. The rationale behind the selection of [N(Tf)_2_] anion was based on its cross-proven high activity in biocatalysis [30,41,42]. It is indeed an example of the conversion of biomass (carbohydrates) into useful chemicals/solvents like ILs.

The first step of the synthesis of [N(CH_3_)_3_GlcOCH_3_][N(Tf)_2_] involved bromination of methyl-α-D-glucopyranoside, wherein CBr_4_ was used as the halogenating agent (Scheme 2). The synthesis was carried out according to the modified procedure, yielding methyl-6-bromo-6-deoxy-α-D-glucopyranoside [49]. The subsequent quaternization of trimethylamine (in ethanolic solution) with the corresponding bromoglycoside was carried out at slight overpressure (in a closed steel reactor). Next, the obtained [N(CH_3_)_3_GlcOCH_3_]Br was used in the anion exchange reaction with Li[N(Tf)_2_], yielding [N(CH_3_)_3_GlcOCH_3_][N(Tf)_2_].

**Figure 1:**
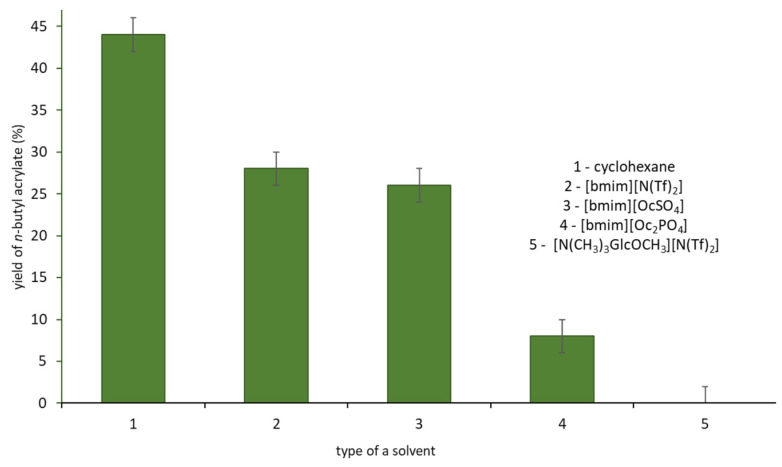
The yield of *n*-butyl acrylate in the esterification of AA with *n*-BuOH in the presence of native CALB in various solvents. *Reaction conditions:* AA 0.072 g (1 mmol), *n*-BuOH 0.149 g (2 mmol), solvent 1 mL, CALB 0.100 g, 24 h, 25 °C, 250 rpm.

Eventually, ILs were used in the model reaction as solvent/catalyst systems (Figure 1). The highest yield (44%) of *n*-butyl acrylate was obtained with cyclohexane as the solvent. All ILs exhibited lower activity in the reaction, up to the complete inhibition for extremely viscous [N(CH_3_)_3_GlcOCH_3_][N(Tf)_2_]. We anticipate that it was caused purely by the high viscosity of ILs, which effectively hindered the mass transport. Nevertheless, believing in the performance of the pre-designed bio-based catalytic systems, and not being discouraged by those results, we attempted to seek alternative strategies, in which physical factors would not exist as leading factors, which can limit the application of IL in biocatalysis. As a result, we have been persevering in using glucose-based IL–ineffective under the above conditions, which is still promising and with a number of other potential benefits, as demonstrated in the initial attempts; we transformed it into the SILP material for CALB adsorption using MWCNTs as the primary support.

### 3.2. Preparation and Characterisation of Biocatalyst CNTs-IL-CALB

**Scheme 3:**
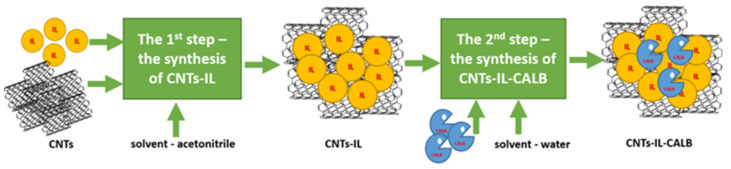
The preparation of the CNTs-IL-CALB biocatalyst.

Based on the series of studies (Supplementary Materials Section S7), [N(CH_3_)_3_GlcOCH_3_][N(Tf)_2_] was pre-selected as the most promising IL for the CALB immobilization on MWCNTs. Several factors influence the SILP properties, including the MWCNTs-IL: CALB ratio and the type of the solvent in the immobilization step. Another important factor is the method of immobilization. In our studies, it was confirmed that the most promising method was based on two stages: first, immobilization of IL on dispersed–in pre-selected solvent–MWCNTs and, second, isolation of SILP via filtering-off, washing, and drying. Subsequently, the immobilization step covered adsorption of CALB from the aqueous SILP dispersions, again followed by filtering-off, washing (with water), and drying (Scheme 3). Cyclohexane in both steps must have been excluded (ESI, Figure S51). In Table 1, the MWCNTs-to-IL mass ratio (from 1:0.1 to 1:1) and the type of solvent (acetonitrile, methanol, THF, cyclohexane) used in the first step of a support formation (i.e., generation of SILP) was studied. For each CNTs-IL pair, the same amount of lipase was applied (7.5 g per 1 g of CNTs-IL) in the second step—synthesis of the biocatalyst. In order to determine the amount of IL and CALB adsorbed on the support, thermogravimetric analysis (TGA) was exploited (Supplementary Materials Section S5). The most active biocatalyst was additionally analyzed via SEM-EDS microscopy (Supplementary Materials Section S4).

Further, as the measure of activity of the obtained CNTs-IL-CALB biocatalysts, the yield of *n*-butyl acrylate obtained in AA esterification process was used (under the identical reaction conditions as in Figure 1). The amount of CNTs-IL-CALB used for the esterification was recalculated from CALB content, fixing it at the same level as in previous experiments (Table 1).

The most active biocatalyst was obtained for the CNTs:IL mass ratio equal to 1:0.5 and acetonitrile as a solvent in the first preparation step. Such SILP in the next step yielded the hybrid material containing 4.2 wt.% CALB content. The lowering of the amount of immobilized IL (from 1:1.0 to 1:0.1 of CNTs:IL per weight) led to higher CALB loadings, while the increase was not linear. Further lowering of immobilized IL led to a slight lowering of the biocatalyst activity. As shown here, the optimum amount of IL was necessary to maintain the highest activity and stability of the enzyme.

**Figure 2:**
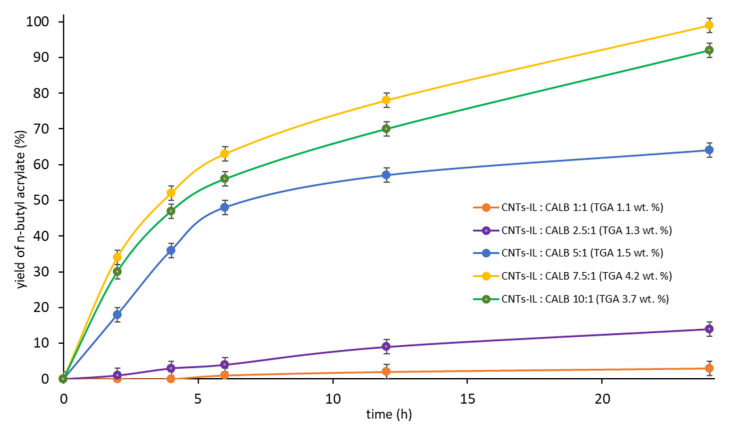
The influence of the mass ratio of CNTs-IL to CALB used for the preparation of CNTs-IL-CALB on the biocatalyst activity. *Preparation of CNTs-IL-CALB conditions*: 1st step: CNTs 1.0 g, [N(CH_3_)_3_GlcOCH_3_][N(Tf)_2_] 0.5 g, acetonitrile 25 mL; 2nd step: CNTs-IL 1.0 g, 1.0–10 g CALB, water 30 mL; *Reaction conditions*: AA 0.072 g (1 mmol), *n*-BuOH 0.149 g (2 mmol), cyclohexane 1 mL, CNTs-[N(CH_3_)_3_GlcOCH_3_][N(Tf)_2_]-CALB 0.150 g, 24 h, 25 °C, 250 rpm.

To confirm that 7.5 g of CALB per 1 g of CNTs-[N(CH_3_)_3_GlcOCH_3_][N(Tf)_2_] is indeed the optimum value for the second step of biocatalyst preparation, some additional tests were performed (Figure 2), in which the mass ratio of CNTs-IL to CALB varied from 1:1 to 1:10. The final content of CALB in the CNTs-IL-CALB (from 1.1 to 4.2 wt.%) was determined by TGA (introduced into the legend).

The activities of five various biocatalysts were compared using the same amount—0.150 g per 1 mmol of AA. As revealed, the most active was the catalytic system containing 4.2 wt.% of CALB. Application of a higher amount of CALB than 7.5-fold mass excess in relation to the support at the immobilization step led to achieving a lower amount of CALB adsorbed on the support, as well as a drop in the overall reaction rate.

Next, screening of the IL structure—from both the cation and anion perspective—toward preparation and activity of the biocatalysts was performed (Table 2). Due to several factors influencing the activity of enzymes in the final biocatalyst, it is impossible to indicate only one of them as playing the crucial role in the interactions. Hence, complex analysis of the physicochemical properties versus an amount of IL adsorbed on the support (in molar and mass context) was necessary. Nevertheless, the amount of IL in the matrix depended mainly on the solubility in acetonitrile and affinity to the support. Prepared biocatalysts were characterized by IL content in a range of from 0.8 to 4.0; thus, the differences were slight. Physical properties (density, viscosity) of IL had a much smaller impact on the activity of CALB after its tethering to the support [30,40,41,52,53]. Among the applied ionic species, ILs based on [N(Tf)_2_] anions and cations containing modified D-glucose (*N*-(6-deoxy-1-*O*-methoxy-α-D-glucopyranosyl)-*N*,*N*,*N*-trimethylammonium and *N*-(6-deoxy-1-*O*-methoxy-α-D-glucopyranosyl)-*N*-butyl-*N*,*N*-dimethylammonium), as well as 1-butyl-3-methylimidazolium, 1-ethyl-1-methylpyrrolidinium cations, and 1-butyl-3-methylimidazolium bis(cyanide)imide, were found to be highly active in the AA esterification (yield of *n*-butyl acrylate 95–99%). Octylsulphates based on dialkylimidazolium cations were also active as CNT modifiers for the synergetic incorporation of CALB, but lowered lipophilicity in 1-ethyl-3-methylimidazolium methylsulphate resulted in a significant drop in activity. The influence of lipophilicity of ILs on the activity of biocatalysts is also visible for the alkylphosphates ILs. The modification of CNTs with 1-butyl-3-methyl bromide or tetrafluoroborate was ineffective. The amount of CALB immobilized on the support varied (0.2–4.2 wt.%) and did not clearly correspond to the type of the IL or its amount adsorbed on the support. In general, lower polarity, lower tendency to formation of hydrogen bonds by anions, lower basicity, proper location in the Hofmeister series (anions and cations), hydrophobicity, or absence of impurities positively affects the lipase stabilization. Thus, in the considered case as in many previous ones, the leading factor remained unclear [30,40,41,52,53].

Furthermore, to clearly demonstrate the advantages of surface modification with IL toward the incorporation of CALB, a comparative experiment using biocatalyst consisting solely of CNTs and CALB (CNTs-CALB) was performed. The mass ratio of CNTs to CALB was fixed at 1:7.5, however the final CALB content in the biocatalyst was very high (16.5 wt.%). The probably highly hydrophobic surface of CNTs display greater affinity to lipase. However, the activity of the biocatalyst was not directly transferred into its activity, as a lower yield of *n*-butyl acrylate (71%) was achieved when compared with the biocatalyst of the same amount of CNTs-[N(CH_3_)_3_GlcOCH_3_][N(Tf)_2_]-CALB (4.2 wt.%). This actually means that even if the biocatalyst contained almost nine times more lipase, it was not as active as the SILP biocatalyst containing [N(CH_3_)_3_GlcOCH_3_][N(Tf)_2_] (yield of *n*-butyl acrylate 99%). In other words, SILP biocatalyst operates far more efficiently for lower lipase loadings. In contrast, an experiment in the presence of CNTs-CALB biocatalyst containing 16.5 wt.% of CALB reduced to 40 mg per 1 mmol of AA—to maintain the same amount of CALB as in 0.150 g of CNTs-[N(CH_3_)_3_GlcOCH_3_][N(Tf)_2_]-CALB—resulted in achieving only a 13% yield of *n*-butyl acrylate within comparable reaction time. This is clear proof for the spectacular influence of ILs on the activity of CALB.

Another important comparison was made with the benchmark biocatalyst Novozyme 435, once again used in the same amount per 1 mmol of AA (0.150 g). The results also demonstrated the outperformance of the hybrid biocatalyst, as it was not as active (yield of product 74%) as CNTs-[N(CH_3_)_3_GlcOCH_3_][N(Tf)_2_]-CALB.

### 3.3. The Influence of the Selected Parameters on the Synthesis of n-Butyl Acrylate

The biocatalyst CNTs-[N(CH_3_)_3_GlcOCH_3_][N(Tf)_2_]-CALB containing 4.2 wt.% of CALB was used for further studies (Table 3). The optimum reaction conditions were found as follows: *n*-BuOH in a two-fold molar excess in relation to the AA and 0.150 g of the biocatalyst per 1 mmol of the AA at 25 °C. The studies toward the determination of the appropriate amount of biocatalyst showed that lower amounts led to a yield decrease. Indeed, amounts of biocatalyst higher than 0.150 g per 1 mmol of the AA most likely inhibited the mass transport, which resulted in reduction of the yield of *n*-butyl acrylate to 90% after 24 h. The experiments also showed that a higher amount than two-fold molar excess of alcohol had a strong impact on the faster deactivation of CALB. Additionally, 25 °C was determined as the optimal temperature for this process. Higher temperatures led to lower yields of the product caused by lower selectivity (presence of the Michael-type addition product).

**Figure 3:**
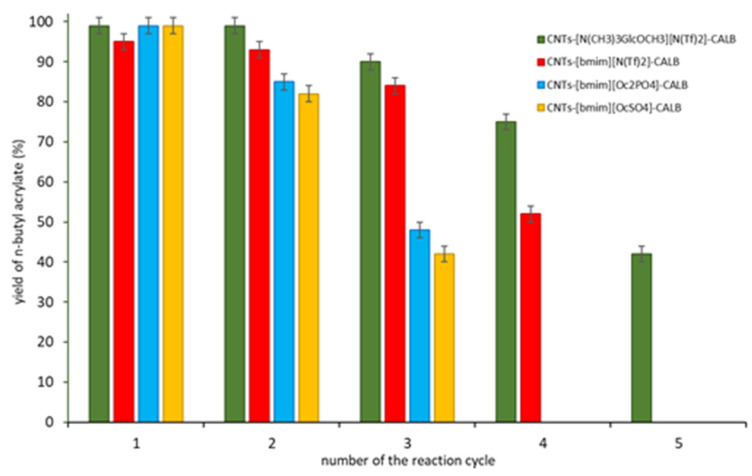
The recycle of CNTs-IL-CALB. *Preparation of CNTs-IL-CALB conditions*: 1st step: CNTs 1.0 g, IL 0.5 g, acetonitrile 25 mL; 2nd step: CNTs-IL 1.0 g, 7.5 g CALB, water 30 mL; *Reaction conditions:* AA 0.072 g (1 mmol), *n*-BuOH 0.149 g (2 mmol), cyclohexane 1 mL, biocatalyst 0.150 g, 24 h, 25 °C, 250 rpm.

Recyclability is the critical aspect in the heterogenous catalysis in batch systems. CNTs-[N(CH_3_)_3_GlcOCH_3_][N(Tf)_2_]-CALB was studied in five subsequent reaction cycles at 25 °C (Figure 3) and compared with other SILP biocatalysts based on commercially available ionic liquids, which allowed obtention of a 99% yield of *n*-butyl acrylate after the first run (CNTs-[bmim][OcSO_4_]-CALB, CNTs-[bmim][Oc_2_PO_4_]-CALB) and with biocatalyst based on privileged for lipases hydrophobic IL–CNTs-[bmim][N(Tf)_2_]-CALB. After each cycle, the catalyst was filtered off, washed, dried, and used for the next reaction cycle. Additionally, the filtrate from each reaction cycle was collected for analysis using the Lowry’s protein detection method, in order to confirm absence of the lipase in the filtrate (Supplementary Materials Section S6). Furthermore, the biocatalysts after the last reaction cycle were analyzed using TGA. Despite the high yield of *n*-butyl acrylate in the first and second reaction cycle (24 h each), its use in the next reaction cycles resulted in reduced yield of the product. This phenomenon can be related to a leaching of IL to the reaction mixture, which was confirmed by TGA analysis (Supplementary Materials Section S5), as well as to the chemical deactivation of the enzyme. Nevertheless, CNTs-[N(CH_3_)_3_GlcOCH_3_][N(Tf)_2_]-CALB was the most stable biocatalytic system.

## 4. Conclusions

We have demonstrated that the pre-designed ternary biocatalyst–encompassing marriage of SILP materials and the mature adsorption technique of enzyme immobilization constituted an up-to-date unrivalled system in the synthesis of *n*-butyl acrylate from acrylic acid and *n*-butanol. The biocatalyst was synthesized from lipase B from *Candida antarctica* incorporated into the bio-based CNTs-glucose ionic liquid hybrid toward yet unprecedented synergetic activity, selectivity, and stability. The overall characteristics of the novel biocatalytic systems were cross-verified and benchmarked against the commercially available biocatalysts, clearly revealing the outperformance of the presented formula. It should be emphasized that the elaborated and developed protocol not only represents a proof-of-concept in biotechnology but, predominantly, opens a new avenue on the route to greener, economic, and superb all-in-one catalysts. In addition, the developed method stands out as a great improvement in comparison with other enzymatic acrylation processes described in the literature (Table 4).

## Figures and Tables

**Table 1:** The influence of the preparation conditions of CNTs-IL-CALB on the biocatalyst activity.

CNTs:IL (g/g)	Solvent ^1^	IL Content in CNTs-IL (wt.%) ^2^	CALB Content in CNTs-IL-CALB (wt.%) ^2^	Yield of *n*-Butyl Acrylate (%) ^3^
1:0.5	**acetonitrile**	**1.8**	**4.2**	**99**
1:0.5	methanol	1.1	4.3	84
1:0.5	THF	2.4	3.1	54
1:1.0	acetonitrile	3.0	1.2	97
1:0.2	acetonitrile	0.8	5.4	91
1:0.1	acetonitrile	0.6	4.4	76

^1^ solvent used at the first step of CNTs-IL-CALB preparation; ^2^ determined by TGA; ^3^ determined by GC; *Preparation of CNTs-IL-CALB conditions*: 1st step: CNTs 1.0 g, [N(CH_3_)_3_GlcOCH_3_][N(Tf)_2_] 0.1–1.0 g, solvent 25 mL; 2nd step: CNTs-[N(CH_3_)_3_GlcOCH_3_][N(Tf)_2_] 1.0 g, 7.5 g CALB, water 30 mL; *Reaction conditions*: AA 0.072 g (1 mmol), *n*-BuOH 0.149 g (2 mmol), cyclohexane 1 mL, CNTs-[N(CH_3_)_3_GlcOCH_3_][N(Tf)_2_]-CALB 0.150 g, 24 h, 25 °C, 250 rpm.

**Table 2:** Influence of the structure of IL on an activity of the biocatalyst.

Structure of the IL/Biocatalyst Name	IL Content in CNTs-IL (wt. %) ^1^	IL Content in CNTs-IL (µmol per 1 g of Support) ^1^	CALB Content in CNTs-IL-CALB (wt.%) ^1^	Yield of *n*-Butyl Acrylate (%) ^2^
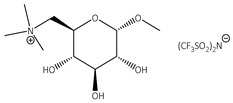 **CNTs-[N(CH_3_)_3_GlcOCH_3_][N(Tf)_2_]-CALB**	**1.8**	**34.4**	**4.2**	**99**
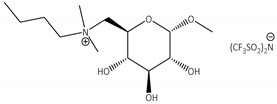 CNTs-[N(CH_3_)_2_(C_4_H_9_)GlcOCH_3_][N(Tf)_2_]-CALB	1.6	28.8	4.2	98
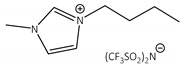 CNTs-[bmim][N(Tf)_2_]-CALB	1.2	28.6	1.2	95
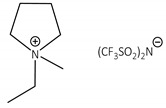 CNTs-[empyrr][N(Tf)_2_]-CALB	0.8	20.3	1.7	95
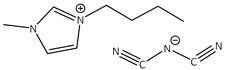 CNTs-[bmim][N(CN)_2_]-CALB	4.0	195.1	0.2	90
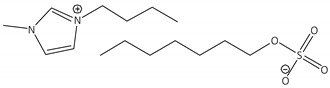 CNTs-[bmim][OcSO_4_]-CALB	2.6	77.8	0.9	99
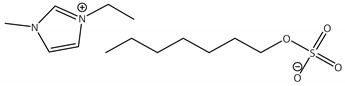 CNTs-[emim][OcSO_4_]-CALB	2.6	81.2	1.6	99
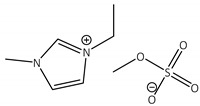 CNTs-[emim][MeSO_4_]-CALB	1.0	45.0	1.4	42
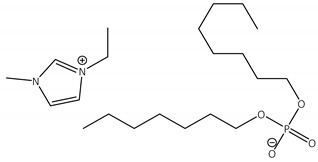 CNTs-[emim][Oc_2_PO_4_]-CALB	1.1	25.5	2.1	99
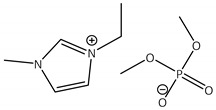 CNTs-[emim][Me_2_PO_4_]-CALB	1.7	72.0	0.7	89
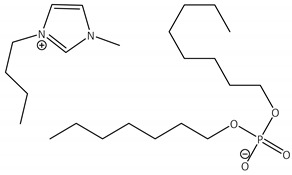 CNTs-[bmim][Oc_2_PO_4_]-CALB	2.9	59.7	0.9	99
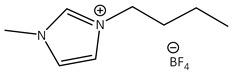 CNTs-[bmim][BF_4_]-CALB	1.1	50.0	1.7	41
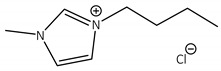 CNTs-[bmim]Cl-CALB	3.3	187.5	0.3	63
CNTs-CALB	-	-	16.5	71
Novozyme 435	-	-	N/A	74

^1^ determined by TGA; ^2^ determined by GC; *Preparation of CNTs-IL-CALB conditions*: 1st step: CNTs 1.0 g, IL 0.5 g, acetonitrile 25 mL; 2nd step: CNTs-IL 1.0 g, 7.5 g CALB, water 30 mL; *Reaction conditions:* AA 0.072 g (1 mmol), *n*-BuOH 0.149 g (2 mmol), cyclohexane 1 mL, CNTs-IL-CALB 0.150 g, 24 h, 25 °C, 250 rpm.

**Table 3:** The influence of the selected parameters on the yield of *n*-butyl acrylate.

Amount of CNTs-IL-CALB (g)	AA:*n*-BuOH Molar Ratio	Temp. (°C)	Yield of *n*-Butyl Acrylate (%) ^1^
0.020	1:2	25	9
0.050	1:2	25	21
0.100	1:2	25	63
**0.150**	**1:2**	**25**	**99**
0.200	1:2	25	90
0.150	1:1	25	92
0.150	1:4	25	42
0.150	1:8	25	23
0.150	1:10 ^2^	25	17
0.150	1:2	15	78
0.150	1:2	45	62

^1^ determined with GC; ^2^ without solvent; *Preparation of CNTs-IL-CALB conditions*: 1st step: CNTs 1.0 g, [N(CH_3_)_3_GlcOCH_3_][N(Tf)_2_] 0.5 g, acetonitrile 25 mL; 2nd step: CNTs-[N(CH_3_)_3_GlcOCH_3_][N(Tf)_2_] 1.0 g, 7.5 g CALB, water 30 mL; *Reaction conditions*: AA 0.072 g (1 mmol), cyclohexane 1 mL, 24 h, 25 °C, 250 rpm.

**Table 4:** The influence of the selected parameters on the yield of *n*-butyl acrylate.

Biocatalyst	Reaction	Yield of the Products (%)	Recycle	Ref.
**CNTs-[N(CH_3_)_3_GlcOCH_3_][N(Tf)_2_]-CALB**	**Esterification of Acrylic Acid with *n*-Butanol**	**99 in 24 h**	**3 Cycles over 90% of Yield of the Product**	**This Work**
Lipase from *Chromobacterium viscosum*	Transesterification of ethyl acrylate with diols (C_3_–C_6_)	26–91 in 2–14 days	n/a	7
Novozyme 435 (CALB immobilized)	Transesterification of methyl acrylate with unsaturated fatty alcohols (C_11_–C_18_)	65–94 in 24 h	n/a	8
Novozyme 435 (CALB immobilized)	Transesterification of ethyl acrylate with *n*-octanol	70	n/a	9
Novozyme 435 (CALB immobilized)	Transesterification of methyl acrylate with bulky alcohols	n/a	n/a	11
Novozyme 435 (CALB immobilized)	Esterification of acrylic acid with *n*-octanol	n/a	n/a	12
Novozyme 435 (CALB immobilized)	Esterification of acrylic acid with β-methylglucoside	n/a	n/a	13

## Data Availability

Data sharing is not applicable for this article.

## Supplementary Materials

The following are available online at https://www.mdpi.com/article/10.3390/ma14113090/s1, Seven sections included in Supplementary Materials: Section S1: GC-FID analysis; Section S2: GC-MS analysis; Section S3: NMR analysis; Section S4: SEM-EDS analysis; Section S5: Thermogravimetric analysis; Section S6: Lowry’s protein detection method; Section S7: Preliminary studies–various ionic liquids.
