# Supplementary material for: Outperformance in Acrylation: Supported D-Glucose-Based Ionic Liquid Phase on MWCNTs for Immobilized Lipase B from Candida antarctica as Catalytic System
Source: Materials (Basel). 2021 Jun 4;14(11):3090. doi: 10.3390/ma14113090 (PMC8200216; doi:10.3390/ma14113090)
Supplement: Supplementary file 1 [file materials-14-03090-s001.zip › materials-1242767-supplementary.pdf]

materials

Article

Outperformance in Acrylation: Supported D-Glucose-Based Ionic Liquid Phase on MWCNTs for Immobilized Lipase B from *Candida antarctica* as Catalytic System

Anna Szelwicka [1], Karol Erfurt [1], Sebastian Jurczyk [2], Slawomir Boncel [3,*] and Anna Chrobok [1,*]

[1] Department of Organic Chemical Technology and Petrochemistry, Silesian University of Technology, Krzywoustego 4, 44-100 Gliwice, Poland; Anna.Szelwicka@polsl.pl (A.S.); karol.erfurt@polsl.pl (K.E.)
[2] Institute for Engineering of Polymer Materials and Dyes, Lukasiewicz Research Network, Sklodowskiej-Curie 55, 87-100 Torun, Poland; sebastian.jurczyk@impib.lukasiewicz.gov.pl
[3] Department of Organic Chemistry, Bioorganic Chemistry and Biotechnology, Silesian University of Technology, Krzywoustego 4, 44-100 Gliwice, Poland
* **Correspondence:** Anna.Chrobok@polsl.pl (A.C.); Slawomir.Boncel@polsl.pl (S.B.); Tel.: +48-32-237-2917 (A.C.); +48-32-237-1272 (S.B.)

Section S1. GC-FID analysis

Table 1. Temperature program using during GC-FID analysis.

Initial temperature (°C)	Increasing rate of the temperature (°C/min)	Holding time (min)
40	-	2
40	20	0
150	45	0
280	-	5

n-butyl acrylate: retention time: 6.22, linear regression equation: y=0.6819x-0.0362, R^2=0.9998

Section S2. GC-MS analysis

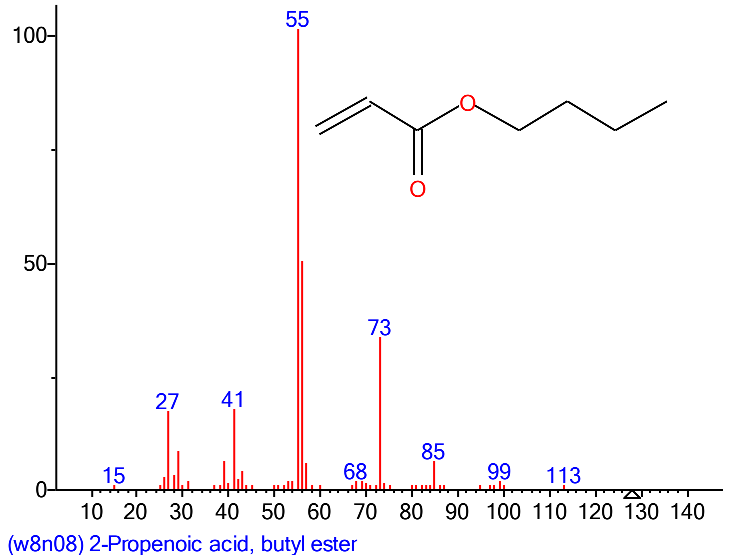

Figure S1. MS spectrum of *n*-butyl acrylate.

Section S3. NMR analysis

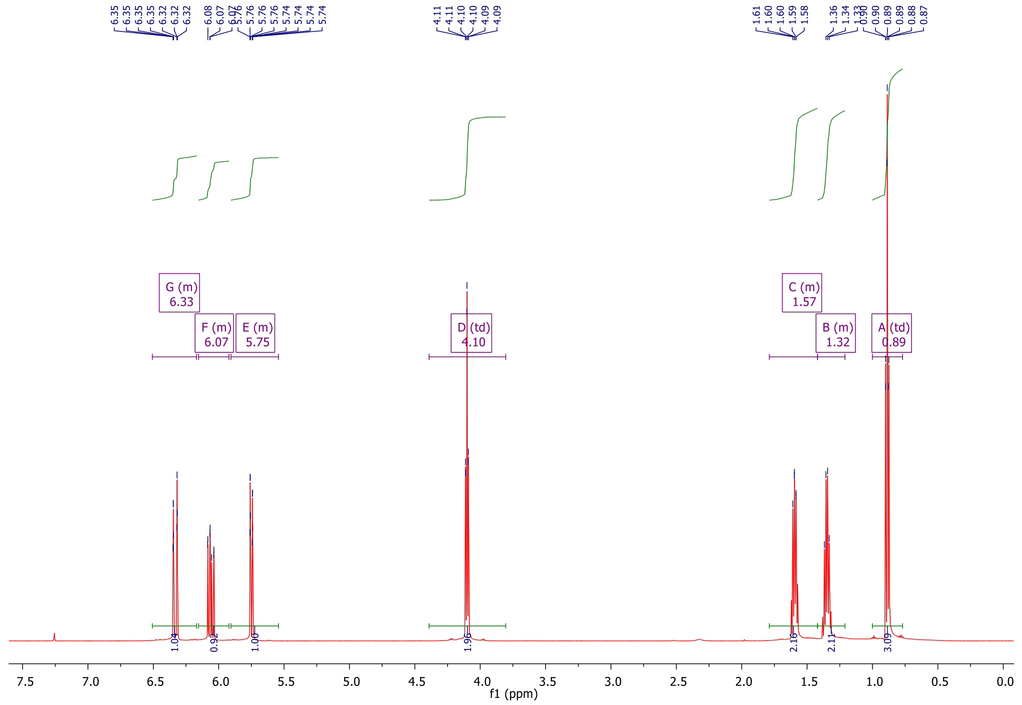

Figure S2. ¹H spectrum of n-butyl acrylate.

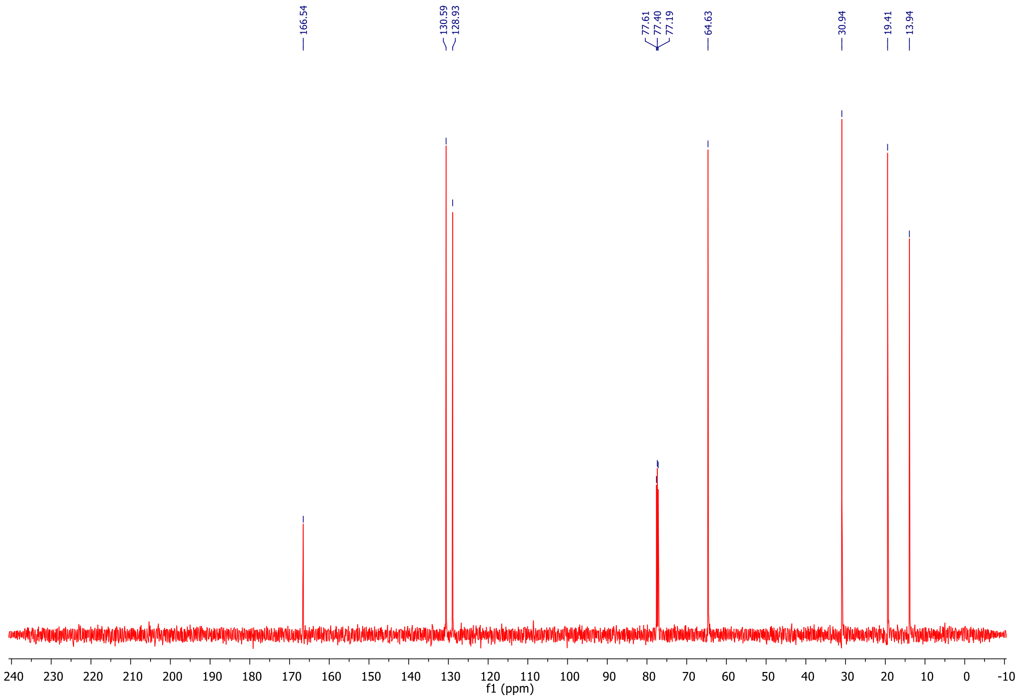

Figure S3. ^{13}C spectrum of n-butyl acrylate.

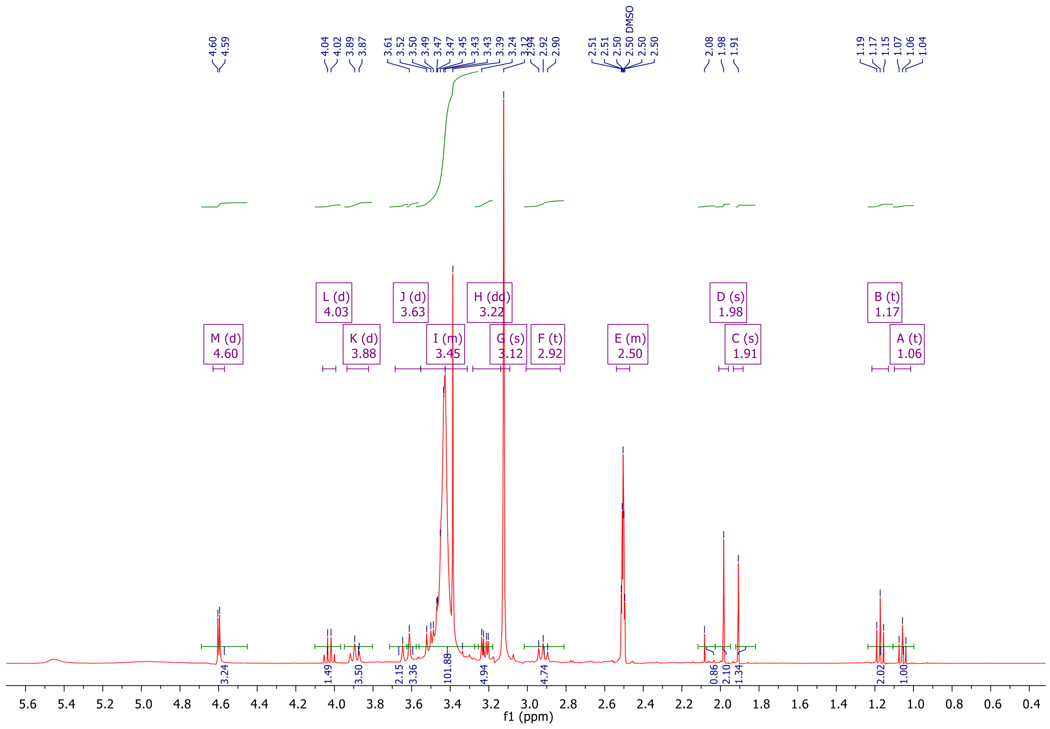

Figure S4. ^{1}H spectrum of $[N(CH_3)_3GlcOCH_3][N(Tf)_2]$.

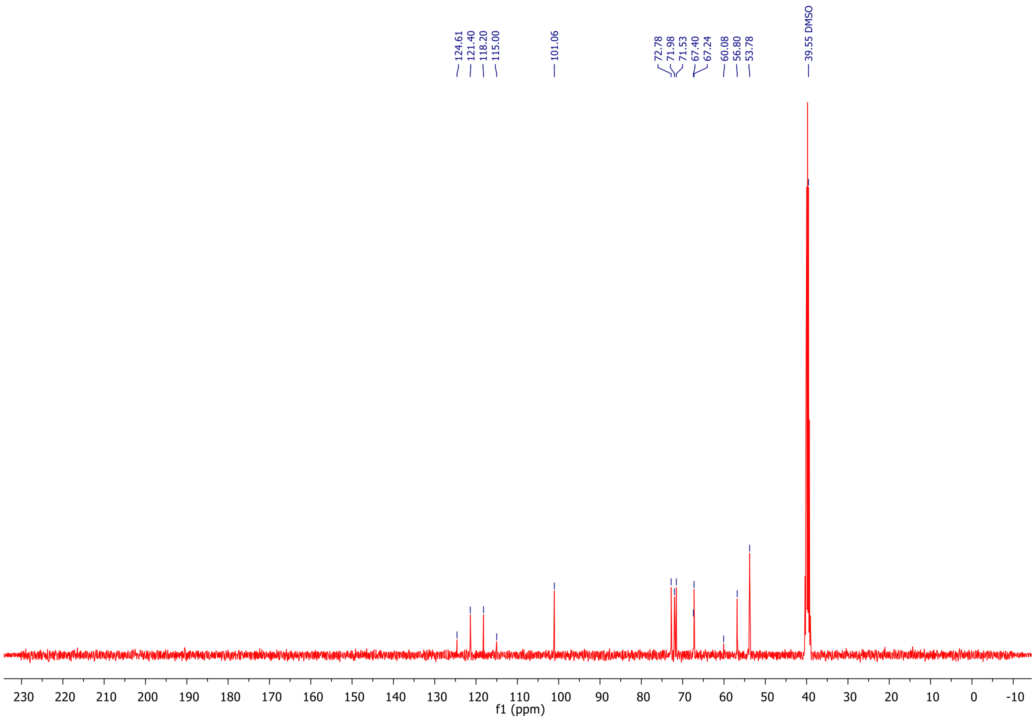

Figure S5. ^{13}C spectrum of $[N(CH_3)_3GlcOCH_3][N(Tf)_2]$.

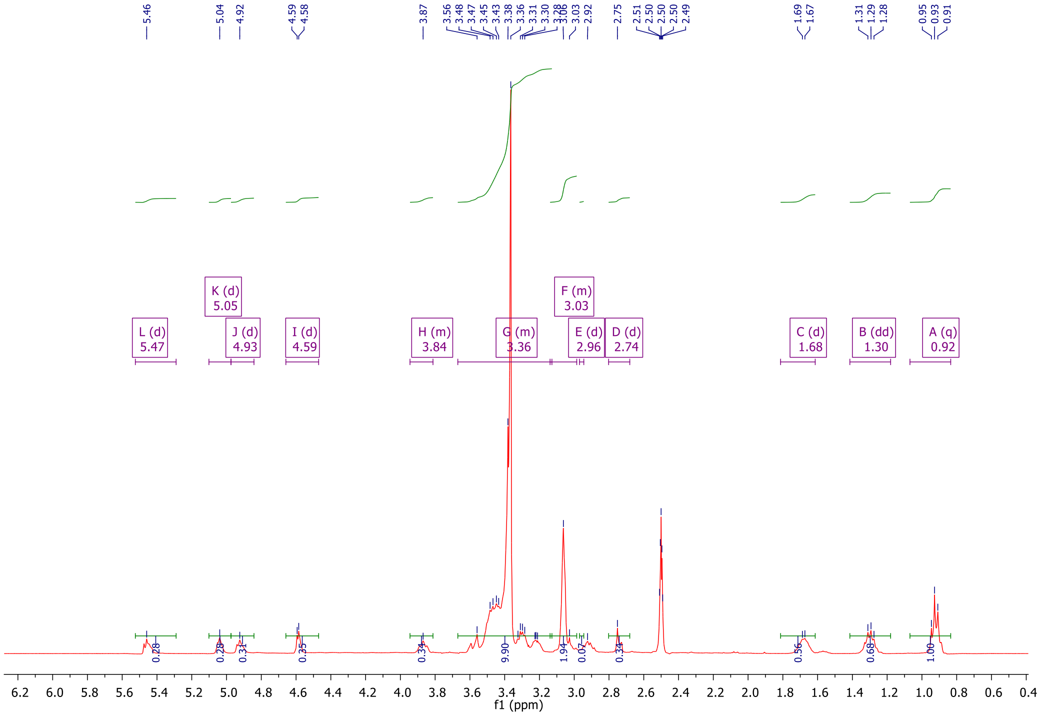

Figure S6. ^{1}H spectrum of $[N(CH_3)_2(C_4H_9)GlcOCH_3][N(Tf)_2]$.

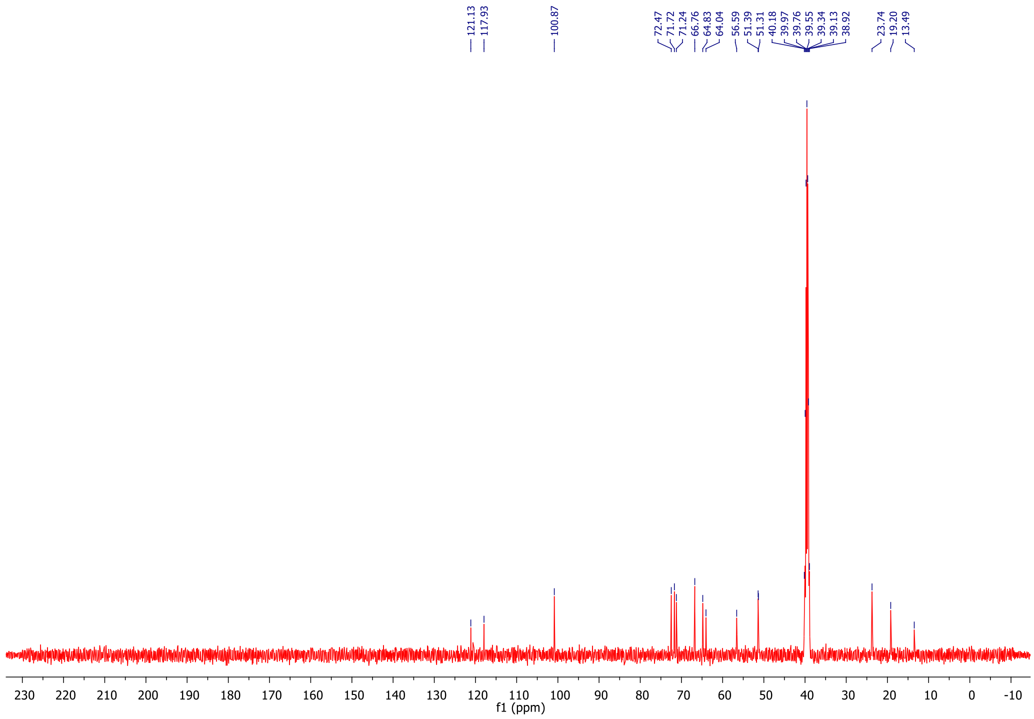

Figure S7. ^{13}C spectrum of $[N(CH_3)_2(C_4H_9)GlcOCH_3][N(Tf)_2]$.

Section S4. SEM-EDS analysis

Scanning electron microscopy (SEM) analysis with an EDS detector was performed for the CNTs-$[N(CH_3)_3GlcOCH_3][N(Tf)_2]$ support (Figure S8). The analyses confirmed the presence of IL on the MWCNT support while morphology of the SILP support emerged as few-micron-size grains composed from entangled nanotubes homogeneously covered with IL. In addition, SEM-EDS analysis was performed for other CNTs-IL supports (Figures S9-S20).

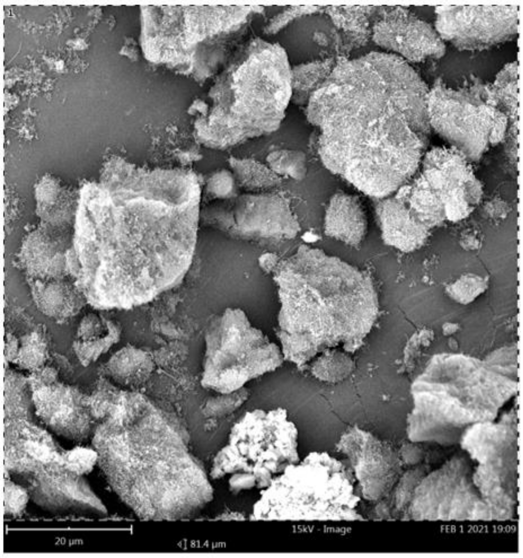

**Element Number**	Element Symbol	Element Name	Atomic Conc.
**6**	C	Carbon	77.06
7	N	Nitrogen	11.22
8	O	Oxygen	10.95

9	F	Fluor	2.01
16	S	Sulphur	0.77

Figure S8. SEM-EDS analysis of CNT-[N(CH_3)$_3$GlcOCH_3][N(Tf)$_2$] support.

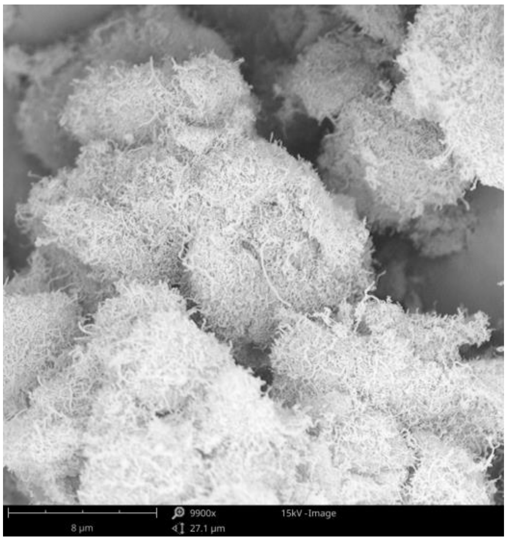

Element Number	Element Symbol	Element Name	Atomic Conc.	Weight Conc.
6	C	Carbon	77.06	72.13
8	O	Oxygen	11.22	14.00
7	N	Nitrogen	10.95	11.96
16	S	Sulphur	0.77	1.92

Figure S9. SEM-EDS analysis of CNTs-[N(CH_3)$_2$(C_4H_9)GlcOCH_3][N(Tf)$_2$].

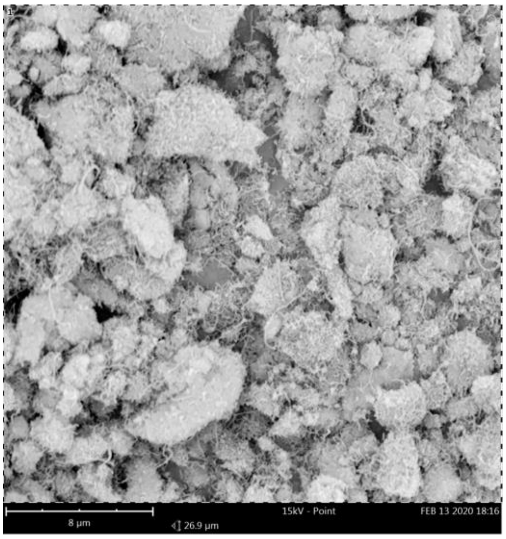

Element Number	Element Symbol	Element Name	Atomic Conc.	Weight Conc.
6	C	Carbon	72.75	66.96
7	N	Nitrogen	12.88	13.82
8	O	Oxygen	9.87	12.10
9	F	Fluorine	3.94	5.74

16	S	Sulphur	0.56	1.38

Figure S10. SEM-EDS analysis of CNTs-[bmim][N(Tf)2].

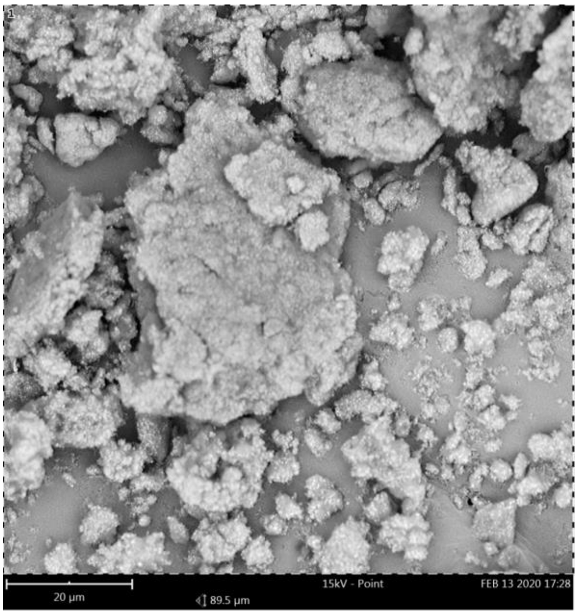

Element Number	Element Symbol	Element Name	Atomic Conc.	Weight Conc.
6	C	Carbon	76.75	71.98
7	N	Nitrogen	11.79	12.90
8	O	Oxygen	10.81	13.50
16	S	Sulphur	0.65	1.63

Figure S11. SEM-EDS analysis of CNTs-[empyrr][N(Tf)2].

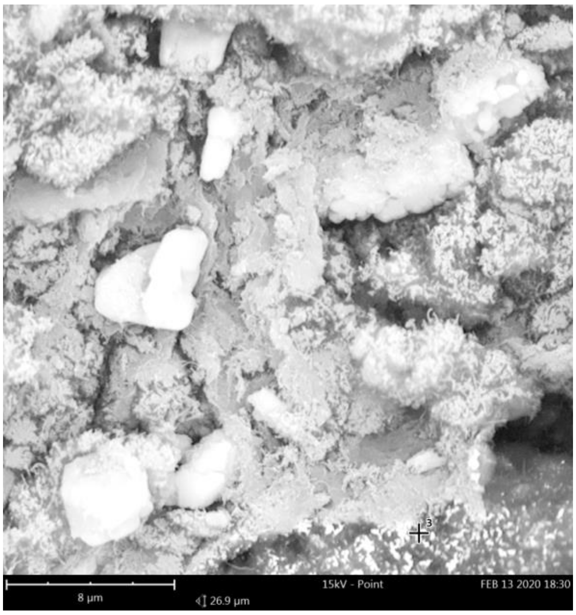

Element Number	Element Symbol	Element Name	Atomic Conc.	Weight Conc.
6	C	Carbon	75.65	72.71

7	N	Nitrogen	24.35	27.29

Figure S12. SEM-EDS analysis of CNTs-[bmim][N(CN)2].

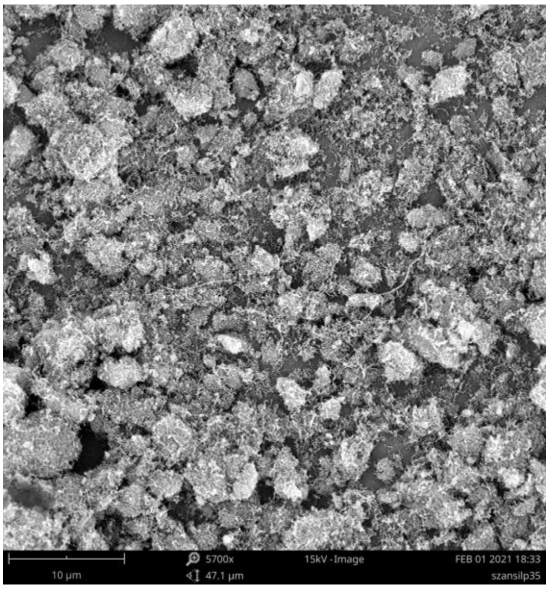

Element Number	Element Symbol	Element Name	Atomic Conc.	Weight Conc.
6	C	Carbon	78.84	74.44
8	O	Oxygen	14.11	17.74
7	N	Nitrogen	7.02	7.73
16	S	Sulphur	0.03	0.09
15	P	Phosphorus	0.00	0.00

Figure S13. SEM-EDS analysis of CNTs-[bmim][OcSO4].

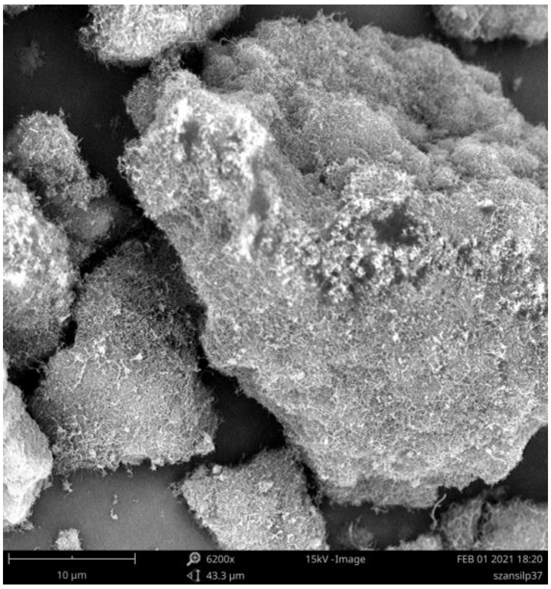

Element Number	Element Symbol	Element Name	Atomic Conc.	Weight Conc.
6.	C	Carbon	81.46	77.26

8	O	Oxygen	13.96	17.63
7	N	Nitrogen	4.55	5.03
16	S	Sulphur	0.03	0.07
15	P	Phosphorus	0.00	0.00

Figure S14. SEM-EDS analysis of CNTs-[emim][OcSO$_4$].

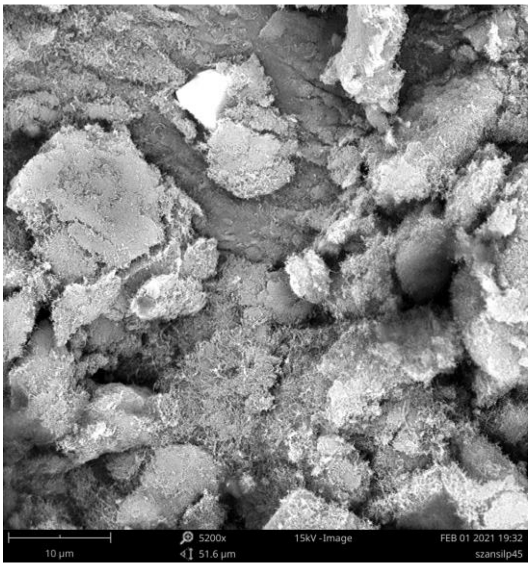

Element Number	Element Symbol	Element Name	Atomic Conc.	Weight Conc.
6	C	Carbon	82.78	78.49
8	O	Oxygen	13.66	17.25
7	N	Nitrogen	3.35	3.70
16	S	Sulphur	0.22	0.55
15	P	Phosphorus	0.00	0.00

Figure S15. SEM-EDS analysis of CNTs-[emim][MeSO$_4$].

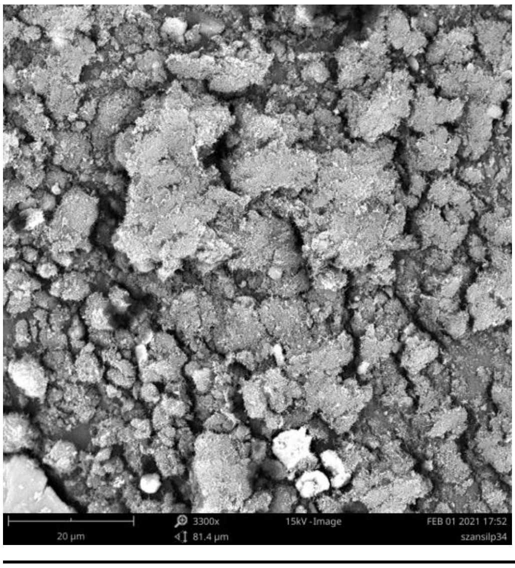

Element Number	Element Symbol	Element Name	Atomic Conc.	Weight Conc.

6	C	Carbon	78.59	74.13
8	O	Oxygen	14.43	18.13
7	N	Nitrogen	6.94	7.63
15	P	Phosphorus	0.03	0.07
16	S	Sulphur	0.02	0.04

Figure S16. SEM-EDS analysis of CNTs-[emim][Oc_2PO_4].

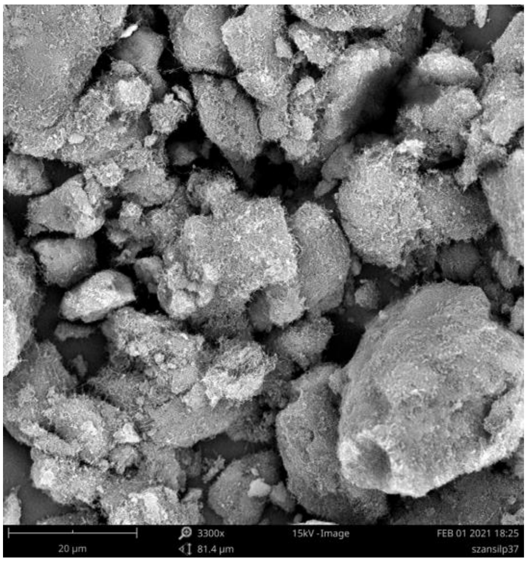

Element Number	Element Symbol	Element Name	Atomic Conc.	Weight Conc.
6	C	Carbon	79.05	74.35
8	O	Oxygen	16.58	20.77
7	N	Nitrogen	4.30	4.71
16	S	Sulphur	0.04	0.10
15	P	Phosphorus	0.03	0.07

Figure S17. SEM-EDS analysis of CNTs-[emim][Me_2PO_4].

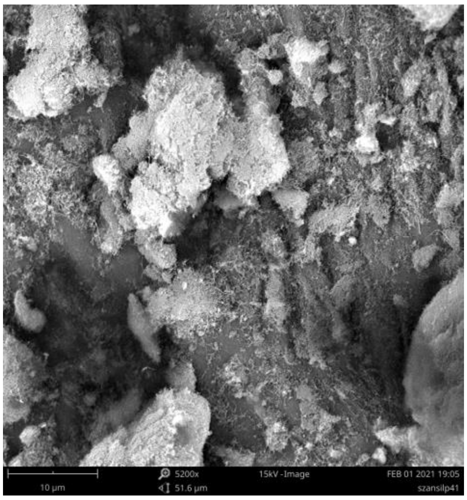

Element Number	Element Symbol	Element Name	Atomic Conc.	Weight Conc.

6	C	Carbon	83.66	80.09
8	O	Oxygen	10.08	12.85
7	N	Nitrogen	6.22	6.94
15	P	Phosphorus	0.04	0.09
16	S	Sulphur	0.01	0.04

Figure S18. SEM-EDS analysis of CNTs-[bmim][Oc_2PO_4].

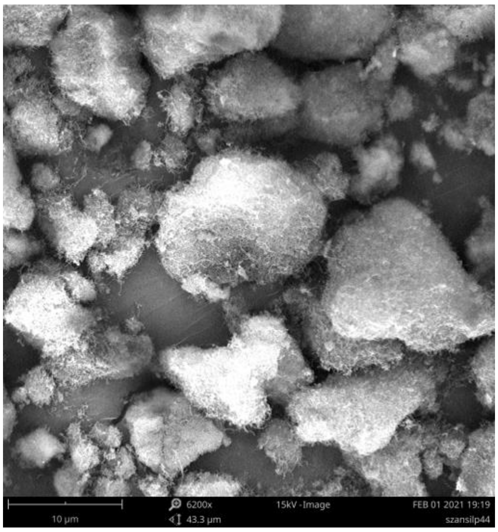

Element Number	Element Symbol	Element Name	Atomic Conc.	Weight Conc.
6	C	Carbon	89.50	87.34
7	N	Nitrogen	6.12	6.97
8	O	Oxygen	4.38	5.70
15	P	Phosphorus	0.00	0.00
16	S	Sulphur	0.00	0.00

Figure S19. SEM-EDS analysis of CNTs-[bmim][BF_4].

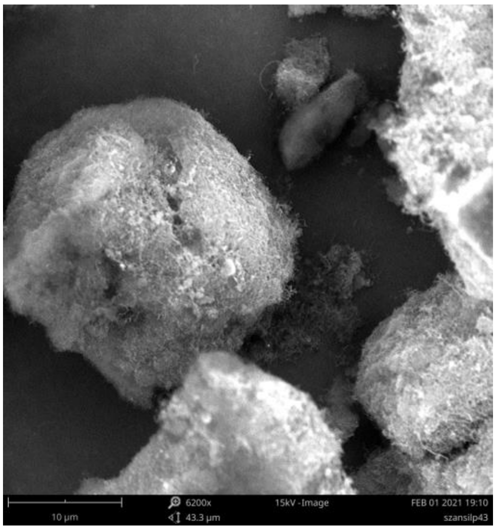

Element Number	Element Symbol	Element Name	Atomic Conc.	Weight Conc.

6	C	Carbon	85.43	82.06
8	O	Oxygen	9.96	12.74
7	N	Nitrogen	4.58	5.13
16	S	Sulphur	0.01	0.04
15	P	Phosphorus	0.01	0.03

Figure S20. SEM-EDS analysis of CNTs-[bmim][Cl].

Section S5. Thermogravimetric analysis

CALB amount was calculated based on a difference between changes of mass of a support and a biocatalyst in a temperature range of 180-450 °C.

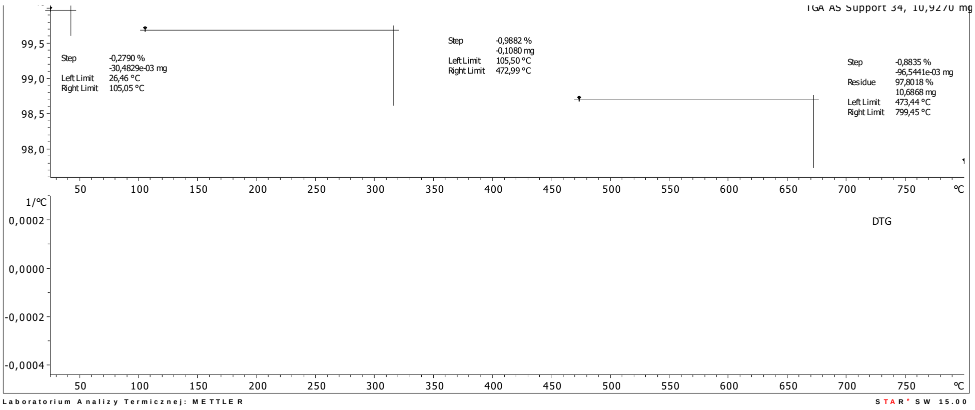

Figure S21. Thermogravimetric analysis of CNTs-[emim][$MeSO_4$].

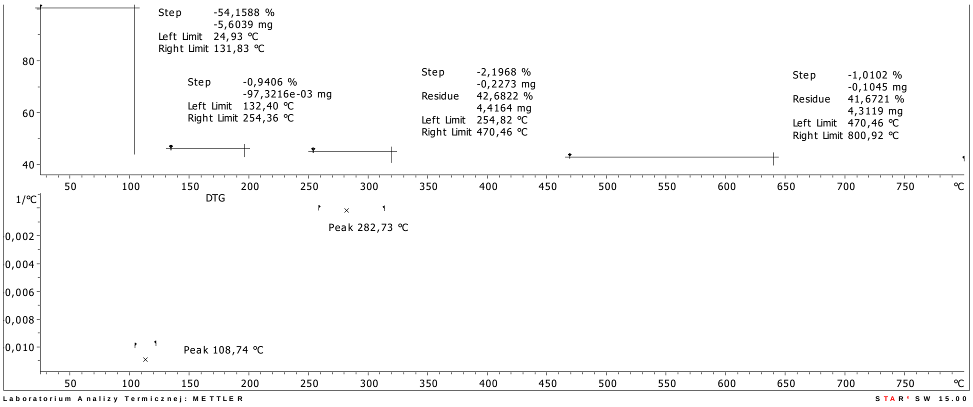

Figure S22. Thermogravimetric analysis of CNTs-[emim][$MeSO_4$]-CALB biocatalyst.

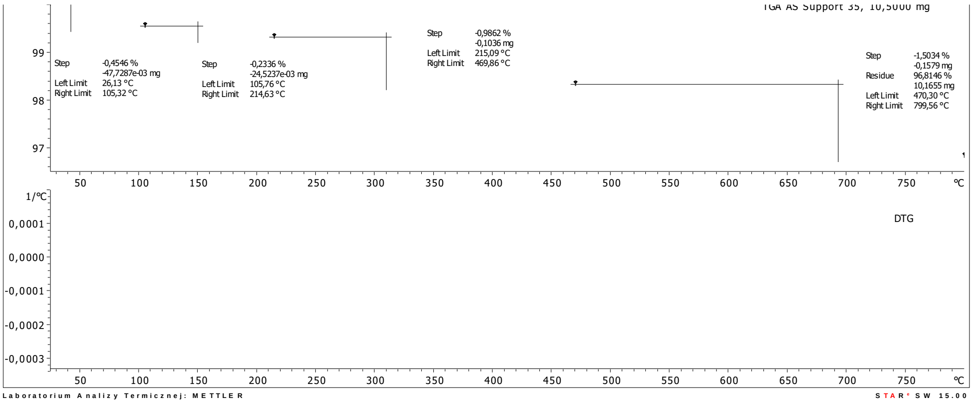

Figure S23. Thermogravimetric analysis of CNTs-[emim][Oc_2PO_4].

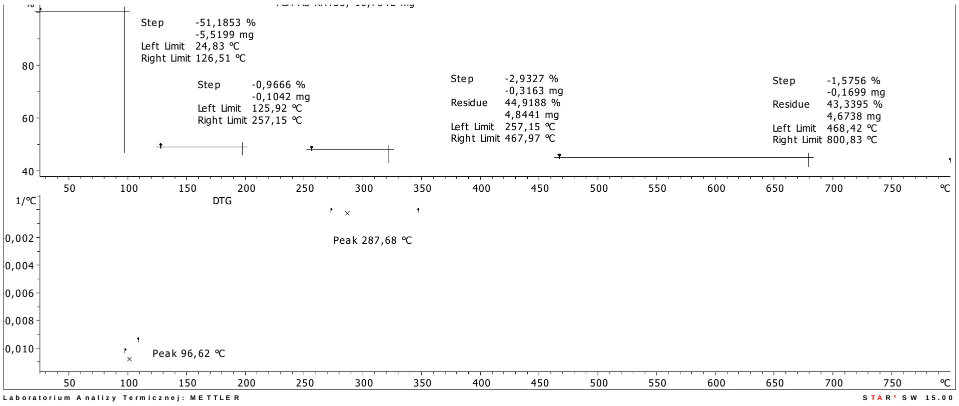

Figure S24. Thermogravimetric analysis of CNTs-[emim][Oc_2PO_4] biocatalyst.

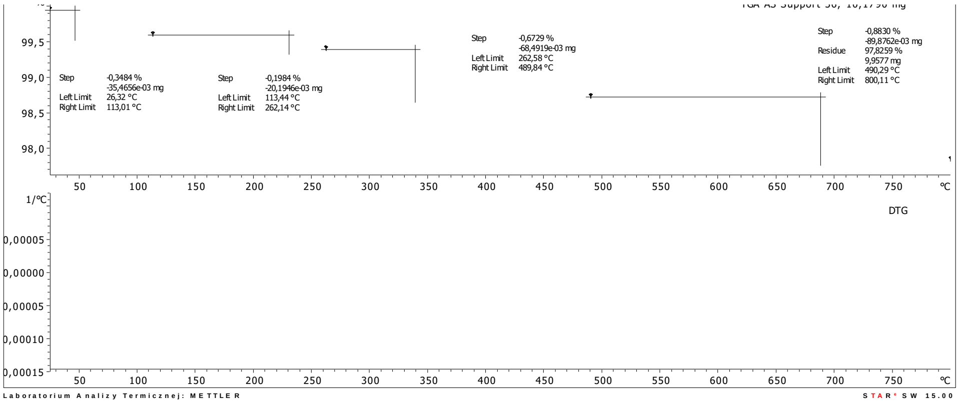

Figure S25. Thermogravimetric analysis of CNTs-[empyrr][$N(Tf)_2$].

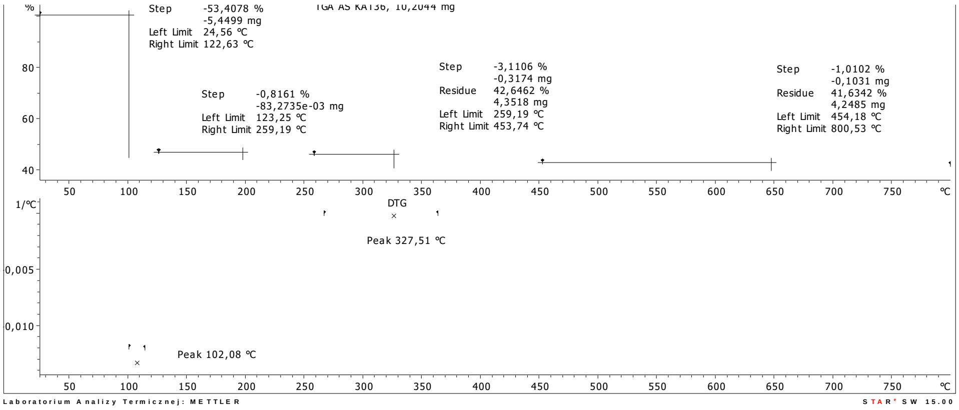

Figure S26. Thermogravimetric analysis of CNTs-[empyrr][$N(Tf)_2$]-CALB biocatalyst.

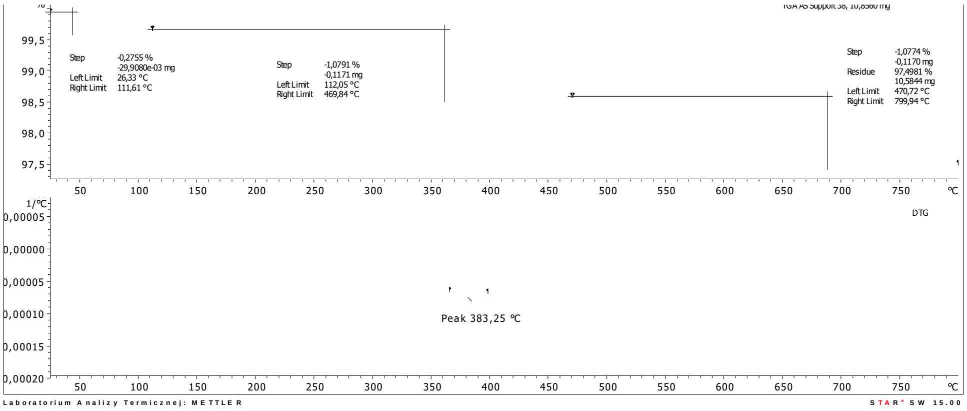

Figure S27. Thermogravimetric analysis of CNTs-[bmim][N(Tf)2].

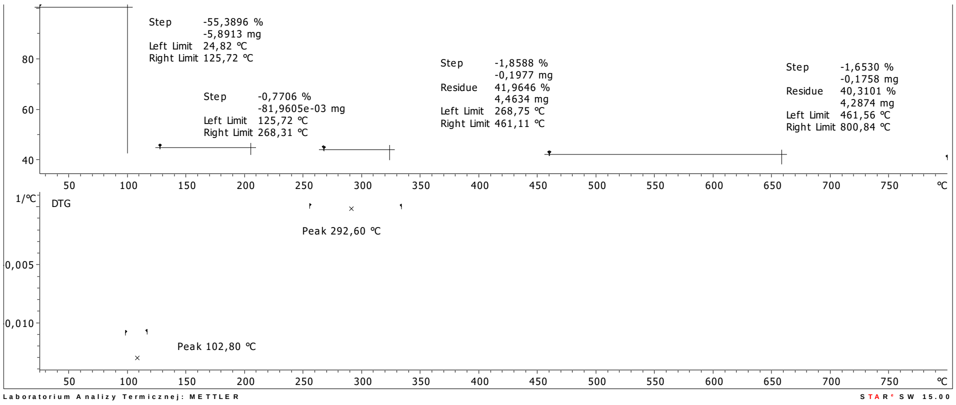

Figure S28. Thermogravimetric analysis of CNTs-[bmim][N(Tf)2]-CALB biocatalyst.

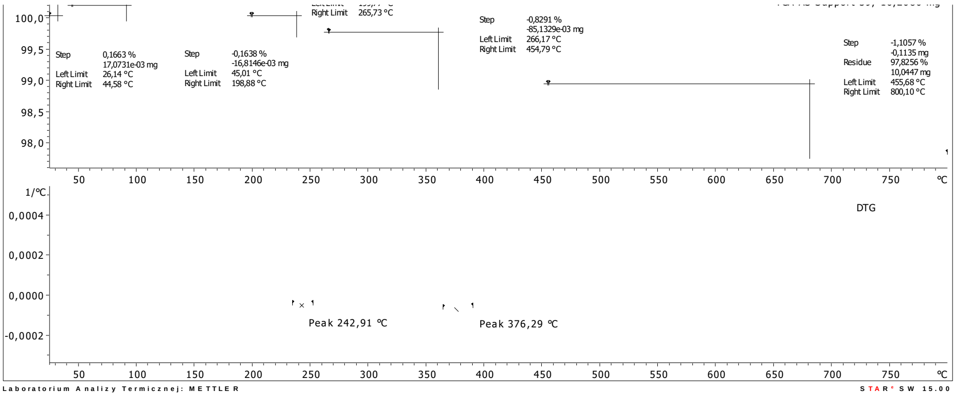

Figure S29. Thermogravimetric analysis of CNTs-[bmim][BF4].

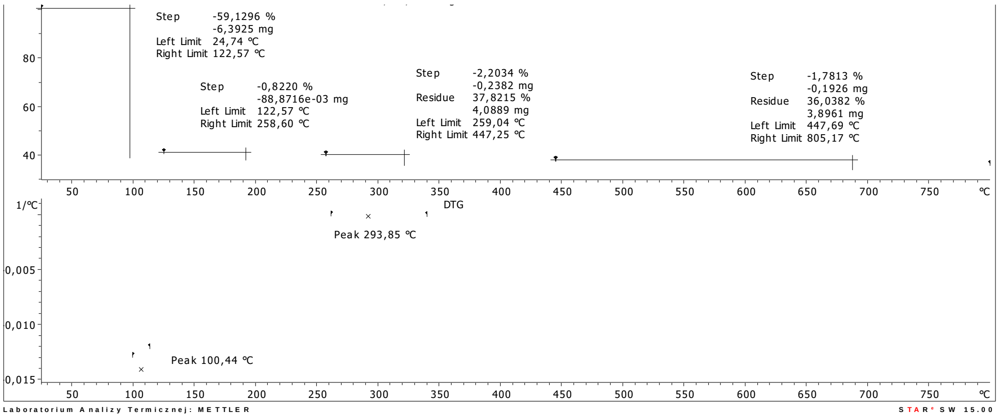

Figure S30. Thermogravimetric analysis of CNTs-[bmim][BF_4]-CALB biocatalyst.

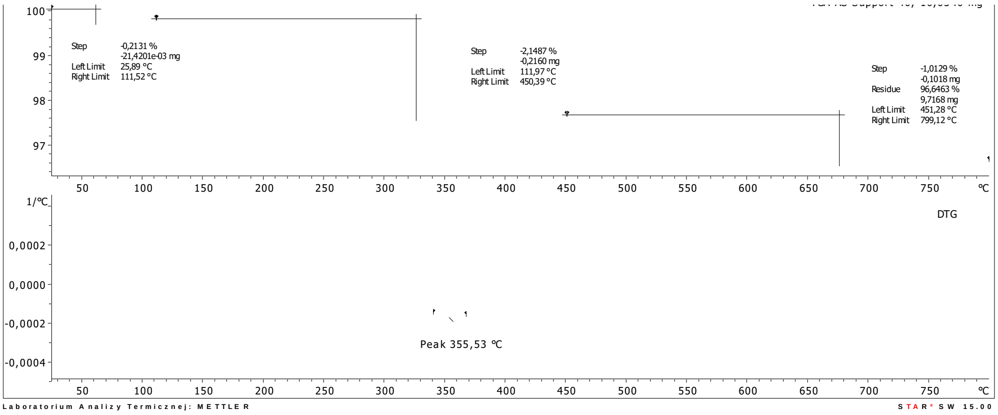

Figure S31. Thermogravimetric analysis of [bmim][$OcSO_4$].

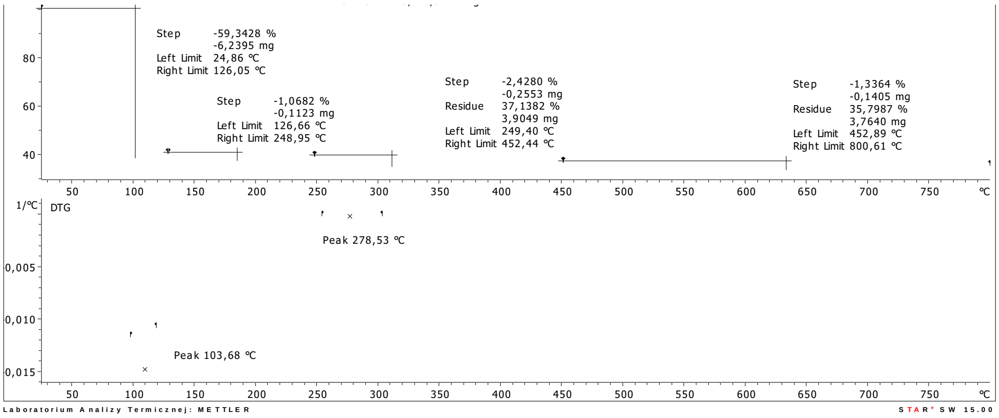

Figure S32. Thermogravimetric analysis of [bmim][$OcSO_4$]-CALB biocatalyst.

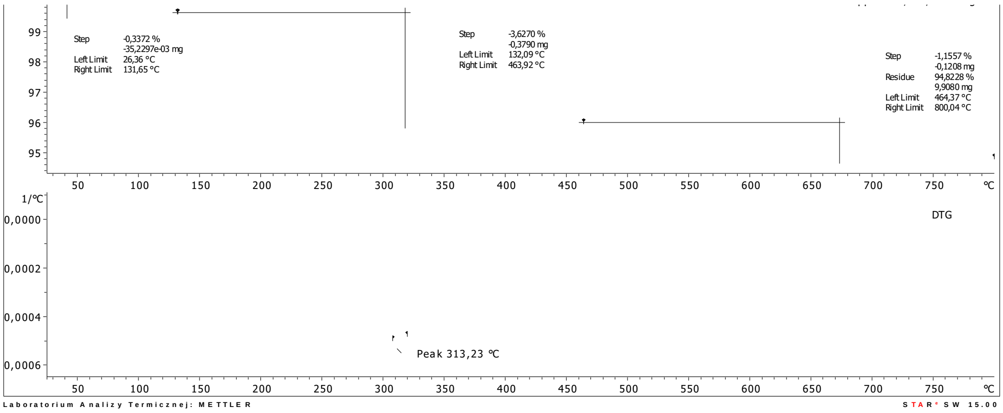

Figure S33. Thermogravimetric analysis of [bmim]Cl.

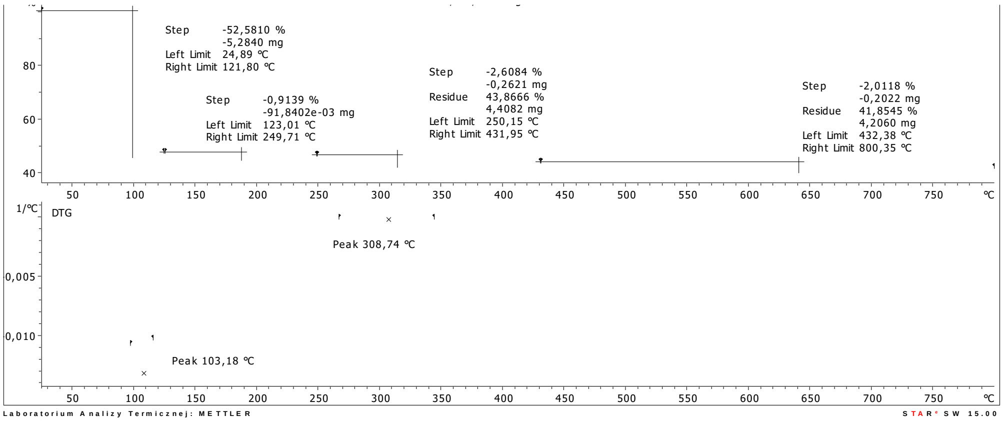

Figure S34. Thermogravimetric analysis of [bmim]Cl-CALB biocatalyst.

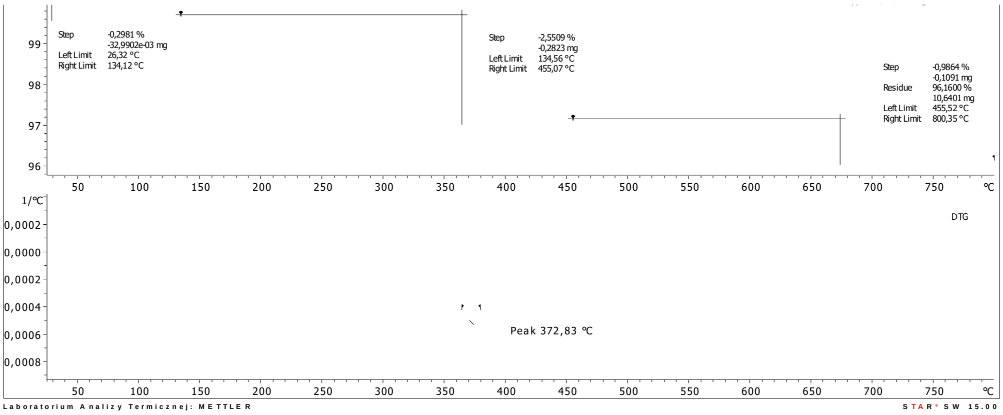

Figure S35. Thermogravimetric analysis of CNTs-[emim][$OcSO_4$].

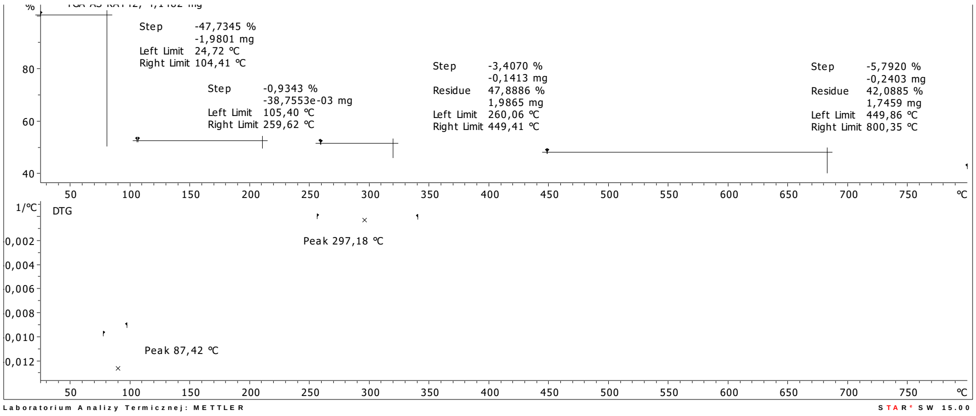

Figure S36. Thermogravimetric analysis of CNTs-[emim][OcSO4]-CALB biocatalyst.

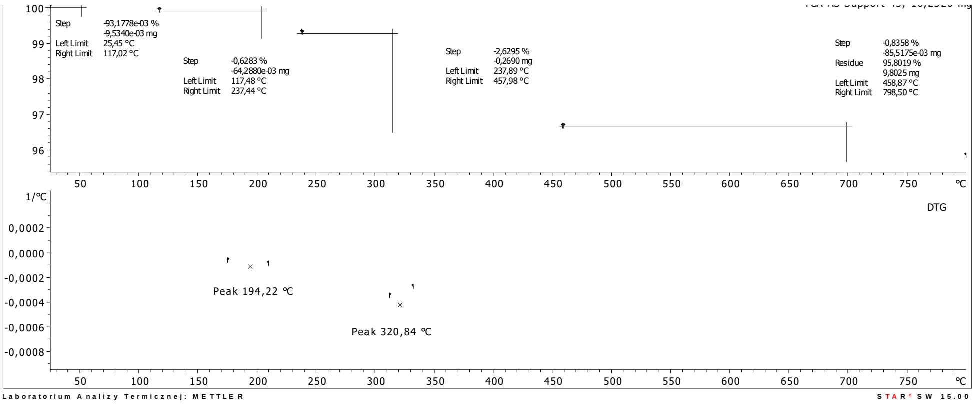

Figure S37. Thermogravimetric analysis of CNTs-[bmim][Oc2PO4].

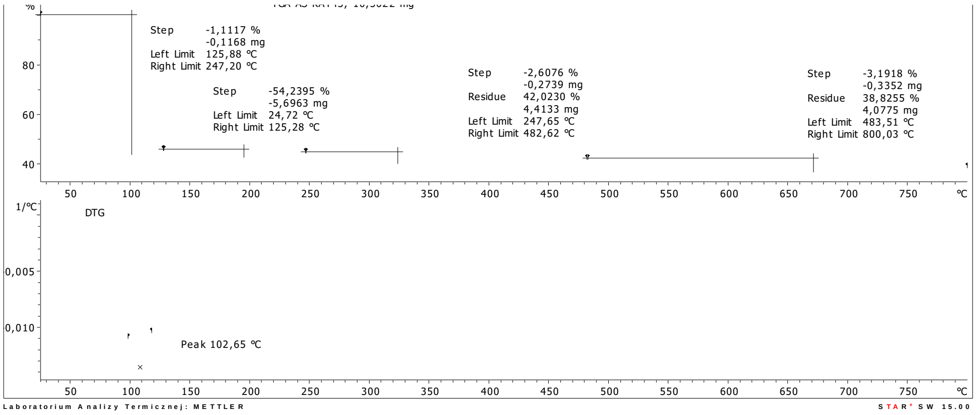

Figure S38. Thermogravimetric analysis of CNTs-[bmim][Oc2PO4]-CALB biocatalyst.

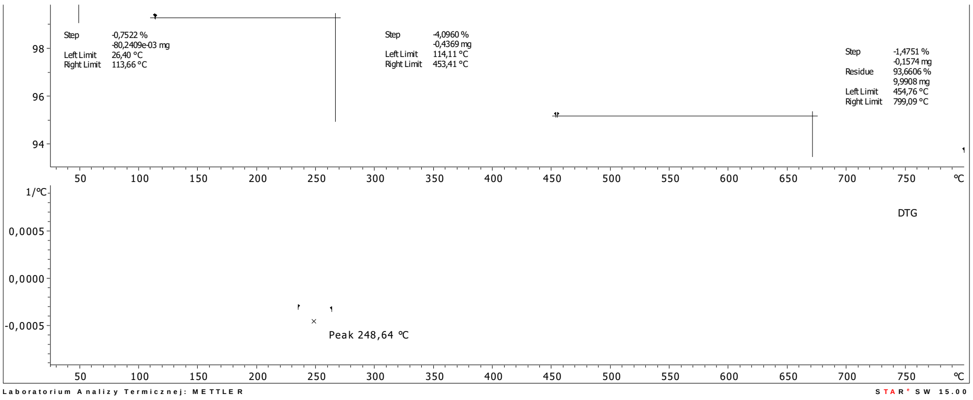

Figure S39. Thermogravimetric analysis of CNTs-[bmim][N(CN)2].

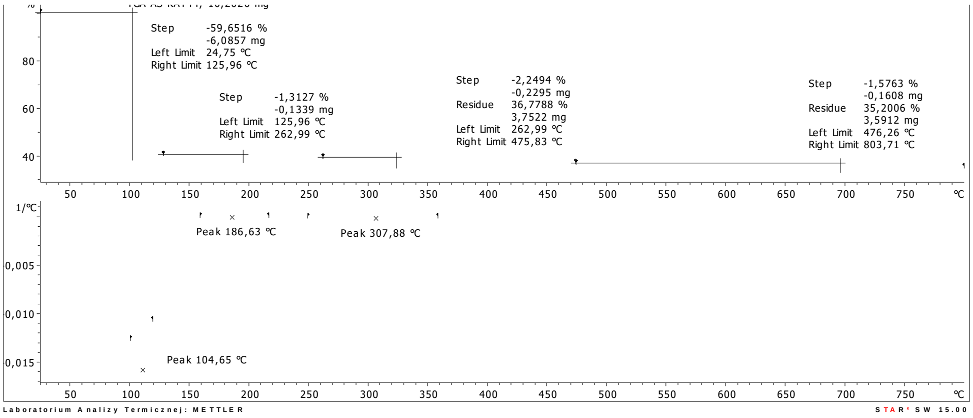

Figure S40. Thermogravimetric analysis of CNTs-[bmim][N(CN)2]-CALB biocatalyst.

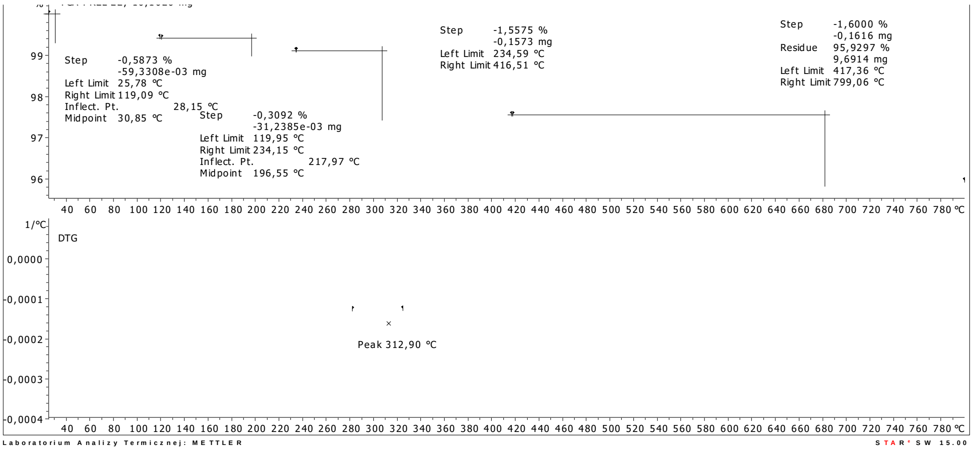

Figure S41. Thermogravimetric analysis of [emim][Me2PO4].

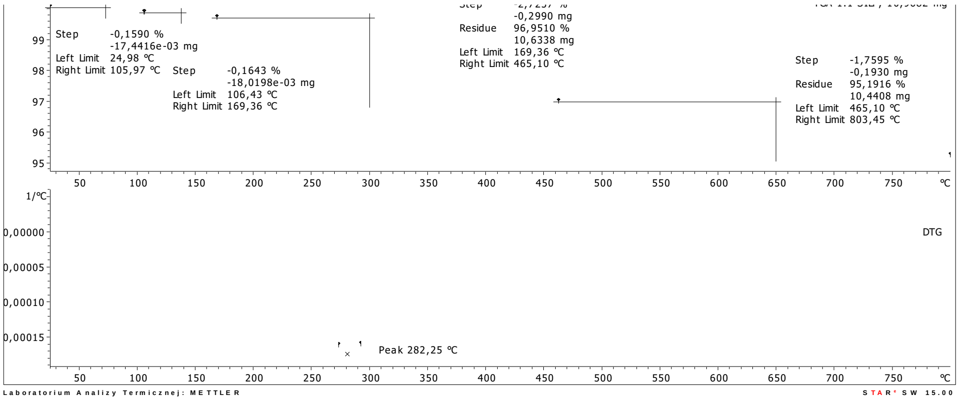

Figure S42. Thermogravimetric analysis of [emim][Me_2PO_4]-CALB biocatalyst.

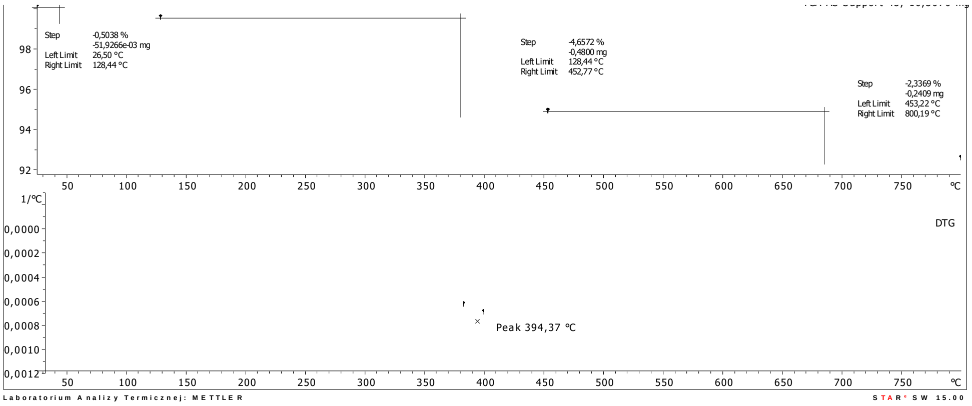

Figure S43. Thermogravimetric analysis of CNTs-[$N(CH_3)_3GlcOCH_3$][$N(Tf)_2$].

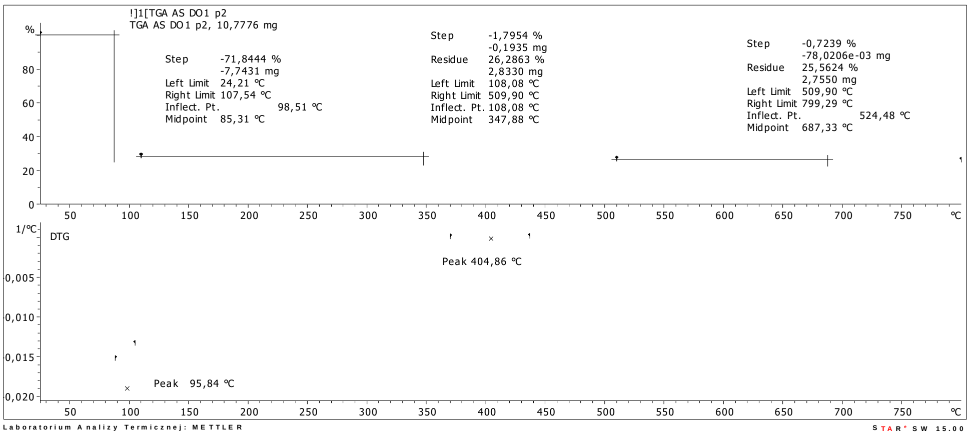

Figure S44. Thermogravimetric analysis of CNTs-[$N(CH_3)_3GlcOCH_3$][$N(Tf)_2$]-CALB biocatalyst.

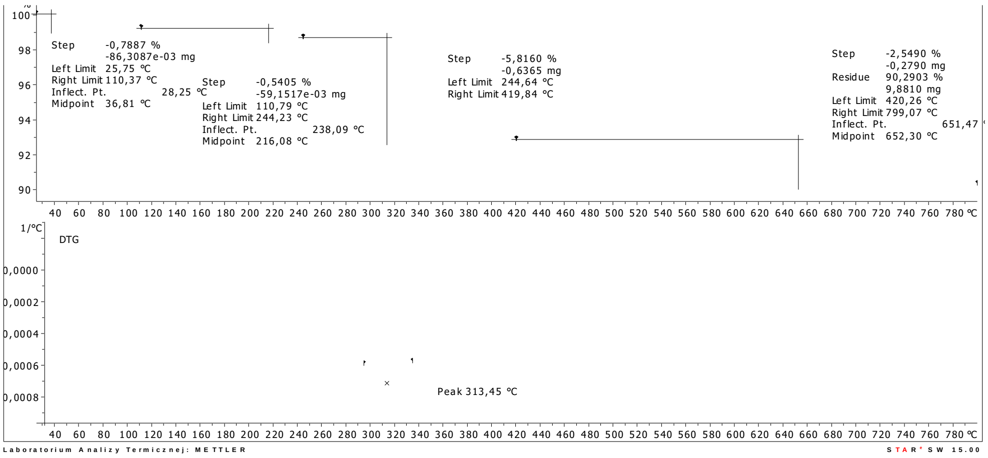

Figure S45. Thermogravimetric analysis of CNTs-[N(CH_3)$_3$GlcOCH_3][N(Tf)$_2$]-CALB biocatalyst – MR CALB: support 1:1.

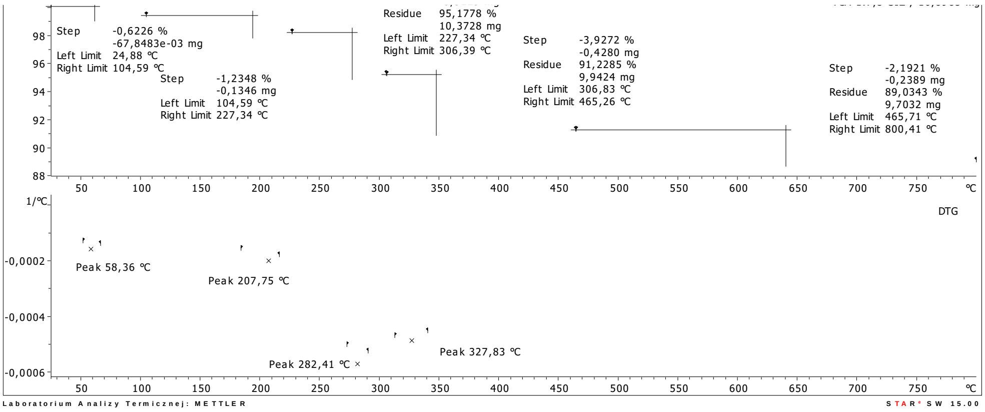

Figure S46. Thermogravimetric analysis of CNTs-[N(CH_3)$_3$GlcOCH_3][N(Tf)$_2$]-CALB biocatalyst – MR CALB: support 2.5:1.

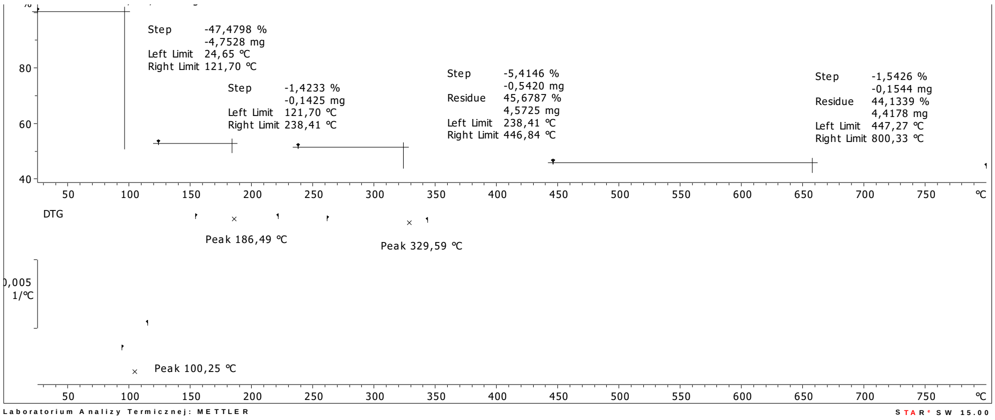

Figure S47. Thermogravimetric analysis of CNTs-[N(CH_3)$_3$GlcOCH_3][N(Tf)$_2$]-CALB biocatalyst – MR CALB: support 5:1.

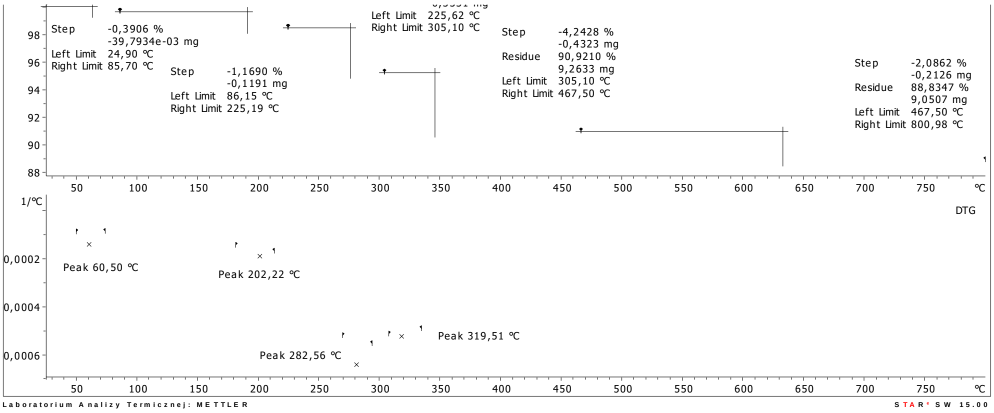

Figure S48. Thermogravimetric analysis of CNTs-[N(CH_3)$_3$GlcOCH_3][N(Tf)$_2$]-CALB biocatalyst – MR CALB: support 10:1.

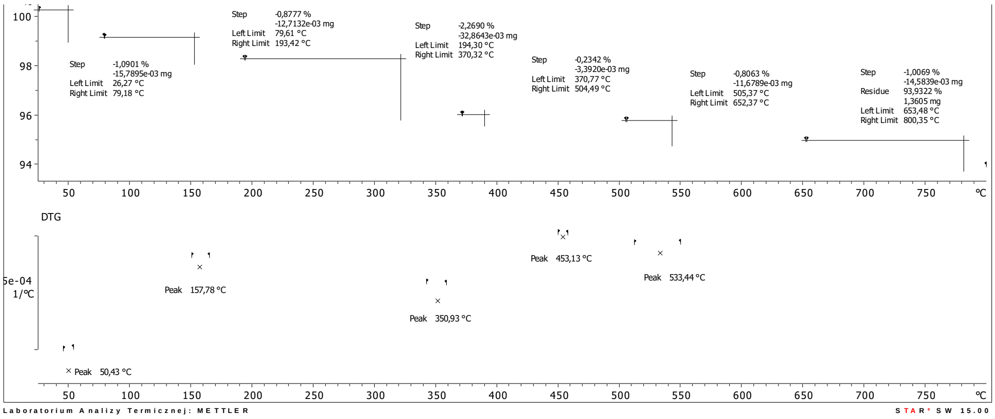

Figure S49. Thermogravimetric analysis of CNTs-[N(CH_3)$_3$GlcOCH_3][N(Tf)$_2$] after stirring in the reaction mixture.

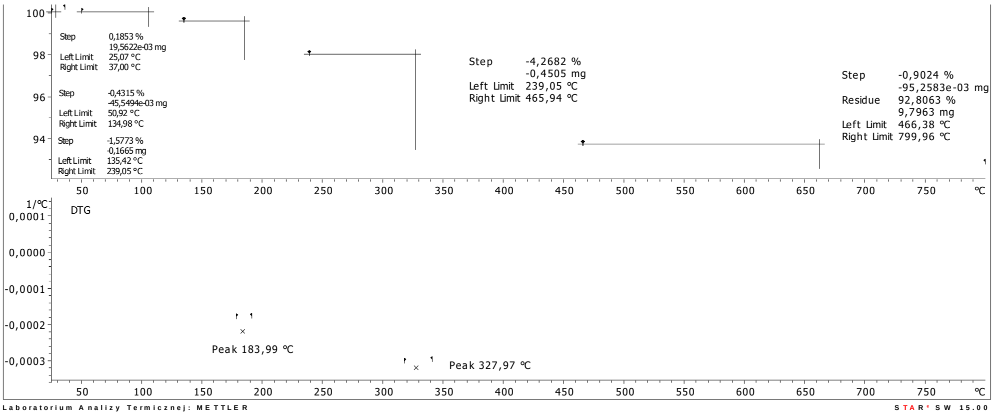

Figure S50. Thermogravimetric analysis of CNTs-[N(CH_3)$_3$GlcOCH_3][N(Tf)$_2$]-CALB biocatalyst after 4th reaction cycle.

Table S2. Estimated amount of IL and CALB after recycle.

Compound	Amount in fresh biocatalyst (wt.%)	Estimated amount after 4th cycle (wt.%)
[N(CH_3)$_3$GlcOCH_3][N(Tf)$_2$] *	1.8	0.9
CALB	4.2	4.1

* experiments without CALB were performed in order to determine the amount of leached IL.

Section S6. Lowry's protein detection method

General procedure of Lowry's protein detection:

Preparation of Folin-Ciocalteu reagent: In a 250 mL-flask, sodium tungstate (12.5 g) and sodium molybdate(VI) (3.13 g) were dissolved in 95 mL of deionised water. Next, 12.5 mL of conc. phosphoric acid(V) and 25 mL of conc. hydrochloric acid were introduced. The mixture was stirred under reflux for 10 h. Next, lithium sulphate (18.5 g) and bromine (approximately 5 drops) were added and the mixture was stirred under reflux for additional 15 min. Then, the mixture was cooling and 125 mL of distilled water was added.

Preparation of alkaline solution: In a 125 mL - flask, 50 mL of a 2% Na_2CO_3 in aqueous NaOH (0.1 M) was prepared. Then, 0.25 mL of an aqueous solution containing 1% $CuSO_4$ and 0.5 mL of 2% sodium tartrate were prepared. 30 min before UV-VIS analysis the solutions were combined to achieve alkaline solution.

Lowry's protein detection method: Into a 25 mL-flask, aqueous-glycerine solutions (3–30 μL/mL) of native lipase B from *Candida antarctica* were added. The calibration curve was calculated *via* UV-VIS analysis of mixtures containing 1 mL of protein solution and 5 mL of the previously obtained alkaline solution, to which 0.5 mL of Folin-Ciocalteu reagent was added after 10 min. Then, the absorbance at a wavelength λ=670 nm was measured. Each measurement was repeated twice and a calibration curve with R2=0.979 was obtained. Next, the aqueous filtrate (after solvent and product evaporation) collected during all reaction cycles was analysed similarly.

Section S7. Preliminary studies – various ionic liquids

For the sake of selection of the most promising IL for the synthesis of SILP biocatalyst, series of experiments concerning an influence of a structure of ILs, suitable conditions for their adsorption on the surface of MWCNTs and type of a solvent applied at the enzyme immobilization step, were tested. Enzyme activity after immobilization was presented as a yield of *n*-butyl acrylate obtained *via* esterification of AA and *n*-butanol (*n*-buOH) after 24 h in a presence of synthesised biocatalysts. In order to determine the elements of the structure of ILs responsible for activation of the enzyme and to design an effective immobilization pathway, 6 selected ILs (with various anions and 1-butyl-3-methylimidazolium cation as well as with various cations and bis(trifluoromethylsulfonyl)imide anion) were taken into considerations: [bmim][BF_4] (IL1), [bmim][$OcSO_4$] (IL2), [bmim][$N(Tf)_2$] (IL3), [bmim][$N(CN)_2$] (IL4), [empyrr][$N(Tf)_2$] (IL5) and [$N(CH_3)_3GlcOCH_3$][$N(Tf)_2$] (IL6).

Figure S51 contains the results of an esterification processes in a presence of synthesised biocatalysts based on ILs 1-6. Supports were prepared in a cyclohexane as a solvent. Water and cyclohexane were taken into considerations for the immobilization of CALB in order to provide the least harmful conditions for the enzyme. Application of water for the immobilization of enzyme allowed to obtain higher yield of *n*-butyl acrylate in the processes carried out in a presence of similar amount of biocatalysts. Beyond the applied ILs with various anions, bis(trifluoromethylsulfonyl)imide led to obtain the most active biocatalyst due to its hydrophobicity. The most active biocatalyst was obtained when [$N(CH_3)_3GlcOCH_3$] [$N(Tf)_2$] was used to modify the MWCNTs.

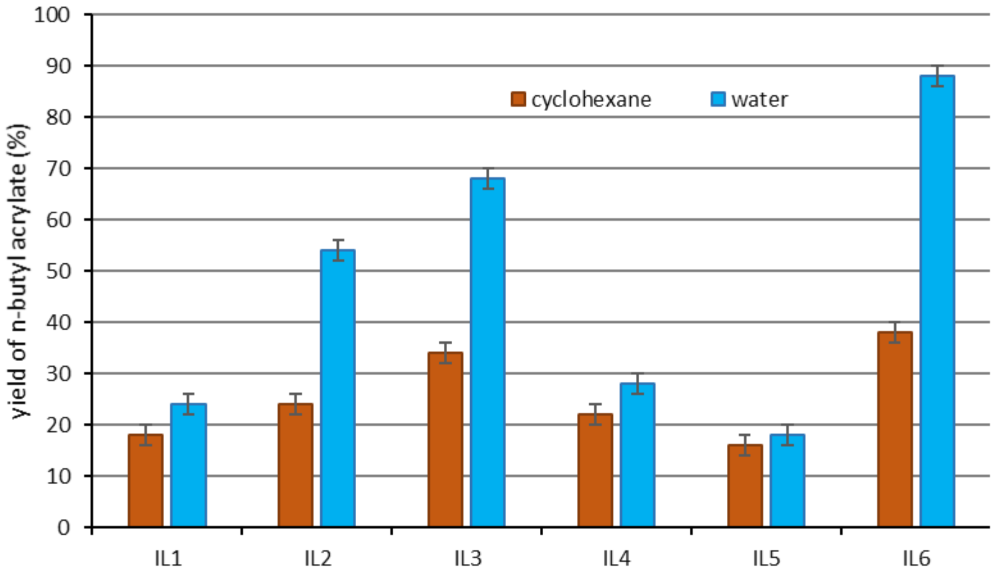

Figure S51. The influence of solvents used for the preparation of CNTs-IL-CALB on the activity of biocatalyst in esterification of AA. In the 1st step of biocatalyst preparation cyclohexane was used for the immobilization of IL, in the second step cyclohexane or water were used for the adsorption of CALB.

Legend: CNTs-[bmim][BF4]-CALB (1), CNTs-[bmim][OcSO4]-CALB (2), CNTs-[bmim][N(Tf)2]-CALB (3), CNTs-[bmim][N(CN)2]-CALB (4), CNTs-[empyrr][N(Tf)2]-CALB (5) and CNTs-[N(CH3)3GlcOCH3][N(Tf)2]-IL (6).

Preparation of CNTs-IL-CALB conditions: 1st step: CNTs 1.0 g, IL 0.5 g, cyclohexane 25 mL; 2nd step: CNTs-IL 1.0 g, 7.5 g CALB, water or cyclohexane 30 mL.

Reaction conditions: AA 0.072 g (1 mmol), *n*-BuOH 0.149 g (2 mmol), cyclohexane 1 mL, CNTs-IL-CALB 0.150 g, 24 h, 25 °C, 250 rpm.
